# Effects of gender-affirming hormones on diurnal cortisol concentrations: A prospective study

**DOI:** 10.1016/j.ynstr.2025.100741

**Published:** 2025-06-18

**Authors:** Margot W.L. Morssinkhof, David Matthew Doyle, Ysbrand D. van der Werf, Martin den Heijer, Annemieke Heijboer, Birit F.P. Broekman, Dirk Jan Stenvers

**Affiliations:** a Department of Medical Psychology, Amsterdam UMC, Amsterdam, the Netherlands; b Department of Psychiatry, Amsterdam UMC, Amsterdam, Vrije Universiteit Amsterdam, the Netherlands; c Department of Endocrinology and Metabolism, Amsterdam UMC, Location University of Amsterdam, Amsterdam, the Netherlands; d Amsterdam Public Health, Amsterdam, the Netherlands; e Department of Anatomy and Neurosciences, Amsterdam UMC, Vrije Universiteit Amsterdam, Amsterdam, the Netherlands; f Amsterdam Neuroscience, Compulsivity Impulsivity and Attention, Amsterdam, the Netherlands; g Endocrine Laboratory, Department of Laboratory Medicine, Amsterdam UMC, Location University of Amsterdam and Vrije Universiteit Amsterdam, Amsterdam, the Netherlands; h Amsterdam Reproduction and Development, Amsterdam, the Netherlands; i Amsterdam Gastroenterology Endocrinology Metabolism, Amsterdam, the Netherlands; j Department of Psychiatry and Medical Psychology, OLVG, Amsterdam, the Netherlands

**Keywords:** Cortisol, HPA axis, Sex hormones, Transgender, Estradiol, Testosterone

## Abstract

The diurnal rhythm of the hypothalamic-pituitary-adrenal (HPA) axis is essential for physical and mental health. There are sex differences in this diurnal rhythm, including steeper diurnal cortisol slopes in females compared to males, and sex hormones likely contribute to this difference. While previous studies found changes in HPA axis responsivity and serum cortisol in transgender people starting gender-affirming hormone therapy (GAHT), no study examined the effect of GAHT on diurnal salivary cortisol. This study examined sex differences in diurnal cortisol and changes in diurnal cortisol after three months of GAHT.

We analyzed salivary cortisol levels in eleven transmasculine (TM) and seven transfeminine (TF) participants before GAHT and after three months of GAHT. Participants collected saliva samples at 30 min, 5.5 h and 10.5 h after awakening, and at bedtime. Absolute cortisol levels and diurnal cortisol slopes were compared between the groups at baseline, and in each group between baseline and three months of GAHT.

Before starting GAHT, the TM group showed a steeper diurnal cortisol slope compared to the TF group. Neither the TM group nor the TF group showed any significant changes in cortisol levels or slopes after GAHT.

We replicate previously reported sex differences in diurnal cortisol levels at baseline, but we find no significant changes in diurnal salivary cortisol after participants start GAHT. This could be associated with homeostatic adaptation of the HPA axis and cortisol-binding globulin concentrations. Future studies should focus on the role of bound and unbound cortisol and stress-related cortisol changes.

## Highlights

• This study examined diurnal cortisol and gender-affirming hormone therapy (GAHT).
• Salivary diurnal cortisol was collected before and after 3 months of GAHT.
• Participants were grouped in transmasculine (TM) and transfeminine (TF) groups.
• TM participants showed a steeper cortisol slope than TF participants before GAHT.
• In both groups, diurnal salivary cortisol did not change after 3 months of GAHT.

## Graphical abstract

**Figure:**
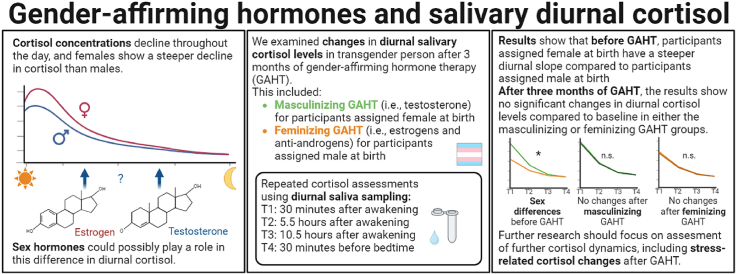

## 1 Introduction

The hypothalamic-pituitary-adrenal (HPA) axis plays an important role in physical and mental health, with cortisol as one of its main outputs. Cortisol is colloquially known as the “stress hormone”, since it has an important role in adaption to stress, but in healthy persons, cortisol levels also fluctuate in a diurnal manner. Cortisol levels are relatively high in the morning and peak around 30–45 min after awakening, which is also called the Cortisol Awakening Response (CAR). Subsequently, cortisol levels typically decrease until bedtime (Pruessner et al., 1997). As a result, cortisol levels follow a diurnal slope, tracking from high cortisol levels in the morning to low levels at bedtime (Adam and Kumari, 2009). This diurnal cortisol rhythm regulates a wide range of processes, including sleep-wake rhythms and metabolic processes, and as a consequence healthy cortisol dynamics are essential for physical and mental health (Adam et al., 2017).

Studies find sex differences in the diurnal cortisol rhythm: females are found to have a higher CAR and a steeper decline in cortisol levels throughout the day (i.e., the diurnal slope) than males (Almeida et al., 2009; Larsson et al., 2009), although findings are still mixed and depend on the methodologies used (Kudielka et al., 2012; Kudielka and Kirschbaum, 2003). Some mixed findings in studies on sex differences in cortisol dynamics point to a role of sex hormones in these sex differences. Several studies find that accounting for oral contraceptive (OC) use, menstrual cycle phase, or menopause status affects findings on diurnal cortisol (Hamidovic et al., 2020; Pruessner et al., 1997; Wolfram et al., 2011). Both endogenous sex hormones (i.e., produced by the body itself) and exogenous sex hormones (i.e., hormonal medication and interventions) could therefore affect cortisol levels. In males, there is a strong time-association between endogenous testosterone and diurnal cortisol: testosterone and cortisol levels are both highest in the morning, and salivary endogenous testosterone and cortisol levels are strongly linked (Crewther et al., 2021; Harden et al., 2016). In females, cortisol levels change throughout the menstrual cycle (Hamidovic et al., 2020): serum cortisol levels are moderately higher in the follicular phase compared to the luteal phase and higher in the premenstrual phase compared to the menstrual phase, especially in morning samples, although salivary cortisol levels have not been found to differ between cycle phases (Hamidovic et al., 2020; Klusmann et al., 2022).

In contrast, there are fewer studies on exogenous hormones in relation to diurnal or resting-state cortisol levels. Two studies found that OC users showed a lower salivary CAR compared to non-users (Bouma et al., 2009; Høgsted et al., 2021), but other studies failed to replicate these effects (Liening et al., 2010; Vibarel-Rebot et al., 2015). In peri- and postmenopausal women, estrogen therapy and combined estrogen and progestin therapy were found to be associated with higher resting-state serum cortisol and higher morning-time serum cortisol as well as with higher salivary cortisol levels at rest (Edwards and Mills, 2008; Gudmundsson et al., 1999).

There is a growing group for whom the effect of sex hormones on diurnal cortisol is relevant, namely transgender people who opt to use gender-affirming hormone therapy (GAHT). GAHT is an important component of gender-affirming care: the overall aim of GAHT is to better align physical presentation with gender identity, in order to reduce gender incongruence and increase gender euphoria. Generally, GAHT can be divided into masculinizing and feminizing GAHT. Masculinizing GAHT generally consists of testosterone, whereas feminizing GAHT generally consists of estrogens and anti-androgens.

There are multiple mechanisms through which sex hormones could affect diurnal salivary cortisol levels. Here we will highlight two mechanisms: changes in HPA axis sensitivity and changes in cortisol-binding globulin (CBG) levels. First, the HPA axis regulates cortisol production through an intricate process of negative feedback loops. The hypothalamus releases corticotropin-releasing hormone (CRH), CRH then stimulates the pituitary gland to release adrenocorticotropic hormone (ACTH) and ACTH then stimulates the adrenal cortex to secrete cortisol. The resulting rise in cortisol levels then inhibits hypothalamic and pituitary neuroendocrine cells, reducing secretion of CRH, ACTH and cortisol through negative feedback loops. Once released and present in blood serum around 90–95 % of serum cortisol is bound to CBG and albumin (Lewis et al., 2005) and not freely available, whereas around 5 % of cortisol is unbound, meaning it is freely bioavailable (Keenan et al., 2004).

Observed sex differences in cortisol levels are reflected in HPA axis regulation: females show increased sensitivity to CRH and ACTH administration, resulting in higher CRH- or ACTH-induced cortisol production compared to males (Gallucci et al., 1993; Uhart et al., 2006). The cause of these differences in HPA axis functioning is not yet clear, but it has been hypothesized that sex hormones could play a role. Notably, OCs were found to increase HPA axis sensitivity to ACTH stimulation, resulting in increased cortisol responsivity In healthy men, suppression of testosterone was associated with lower ACTH but higher cortisol levels in response to CRH administration (Rubinow et al., 2005).

In studies on cortisol and sex hormones, it is important to make a distinction between the measurement of unbound and bound cortisol. In serum, cortisol measurements often reflect the level of total cortisol, which means that the fractions of unbound and bound cortisol are combined (El-Farhan et al., 2017), albeit that this is only fully the case in well-developed cortisol methods (Vos et al., 2017). In saliva, however, assays most commonly only reflect the unbound (i.e., free, bioavailable) fraction of cortisol, without the cortisol bound to CBG and albumin (El-Farhan et al., 2017; Umeda et al., 1981). Estrogens, both exogenous and endogenous forms, increase the rate of CBG synthesis (Özcan et al., 2024; van der Vange et al., 1990), the magnitude depending on administration form and substance. A recent prospective study found that CBG increased after 12 months of feminizing GAHT, with oral estrogens resulting in a higher CBG level than transdermal estrogens, and CBG decreased after 12 months of masculinizing GAHT (Stangl et al., 2025).

Thus far, three studies have examined the effects of GAHT on cortisol, although they mostly focused on HPA axis stimulation and serum cortisol (see Table 1 for a summary of study setups and results). In 2006, Mueller et al. examined the effects of 12 and 24 months of feminizing GAHT, consisting of Gonadatropin-Releasing Hormone (GnRH) analogues combined with oral estradiol, on serum cortisol and CBG levels in a sample of 40 participants who started feminizing GAHT. Serum cortisol levels and CBG levels increased significantly after both 12 and 24 months of feminizing GAHT, although they did not report the fraction of free cortisol. Second, Fuss et al. (2019) assessed the effects of three months of GAHT use on HPA axis dynamics in 15 participants who started masculinizing GAHT and 10 participants who started feminizing GAHT. The authors conducted a dexamethasone/CRH-test before starting GAHT and after three months of GAHT: at both time points they suppressed HPA axis activity, then administered CRH and measured resulting ACTH and cortisol levels in blood. After three months of masculinizing GAHT, morning cortisol levels before CRH administration were lower, and ACTH and cortisol secretion post-CRH were higher compared to baseline. After three months of feminizing GAHT, morning cortisol levels before CRH administration were higher, while ACTH and cortisol secretion post-CRH were lower compared to baseline. These authors did not report free cortisol concentrations either. Lastly, Sofer et al. (2024) examined serum and saliva cortisol in 10 participants starting masculinizing GAHT and 18 participants starting feminizing GAHT at baseline and after ACTH stimulation test at two time points, one of which before GAHT and the other after six months of GAHT. After six months of masculinizing GAHT, there was a reduction in serum total cortisol response, serum free cortisol response and salivary cortisol response post-ACTH administration. After six months of feminizing GAHT, there were no significant changes in total cortisol or free cortisol and a reduction in salivary cortisol post-ACTH stimulation.

**Table 1:** Studies on GAHT and cortisol.

Study	Mueller et al. (2006)	Fuss et al. (2019)	Sofer et al. (2024)
**Participants**	40 TF participants	13 TM participants and 8 TF participants	10 TM participants and 8 TF participants
**GAHT**	TF: 6 mg oral estradiol & 3.8 mg goserelin acetate	TM: Testosterone undecanoate 1000 mg every 12 weeks TF: Estradiol (oral or gel) & cyproterone acetate (dose range 2.5 mg–25 mg)	TM: Testosterone TF: Oral estradiol treatment & spironolactone or cyproterone acetate
**Follow up**	12 and 24 months of GAHT	Three months of GAHT	Six months of GAHT
**HPA axis assessment**	Serum (8am–10am) No dynamic assessment	Baseline serum (8am) Post-dexamethasone serum (8am, next day) Intravenous administration of CRH → post-CRH serum (+30, +45, +60, +75 min)	Baseline serum and saliva (8am–10am) Intravenous administration of ACTH → Post-ACTH serum and saliva (+20, +30, +40)
**Outcomes and assays**	**Cortisol** measured with competitive immunoassay (Immulite, 2000, Diagnostic Products Corp., Los Angeles, California, USA). The intra−assay CVs were 5.2 %, 5.3 % and 6.2 % at the levels of 234.5, 496.6 and 717.3 nmol/l at interassay CVs of 6.8 %, 7.2 % and 7.3 %, respectively. **CBG** was measured with an radio−immunometric assay (AMP, Obrigheim, Germany). The intra−assay CVs were 3.9 % and 2.9 % at the levels of 4.26 and 108.0 g/ml at interassay CVs of 5.5 %, and 2.4 %, respectively.	**Cortisol** measured by radioimmunoassay (DRG International, Inc., U.S.A.), intra-assay CV of 8.6 % and interassay CV of 10.8 %. **CBG** measured by radioimmunoassay (DRG International, Inc., U.S.A.), intra-assay CV of 6.2 % and interassay CV of 8.7 %.	**Serum total cortisol** was measured with an electrochemi-luminescence immunoassay (Cobas 411; Roche). The intra- and inter-assay coefficients of variation were 1.4 % and 1.6 %, respectively. **Serum free cortisol** was measured using the same assay (Cobas) following equilibrium dialysis (Limor et al., 2011). Coefficients of variation were not reported. **Salivary cortisol** was measured using a modification of the RIA (Coat-a-Count; Diagnostic Products), with an intra-assay CV of 5.1 % and inter-assay CV of 4 %.
**Results in transmasculine group**	–	**Baseline serum cortisol** decreased from 204.9 ± 46.8 μg/dl at baseline to 175.5 ± 36.7 μg/dl after 3 months of GAHT, showing a 14 % reduction. **CRH-stimulated cortisol** decreased from 2045.9 ± 1318 μg/dl × min at baseline to 1202.9 ± 363.7 μg/dl × min after 3 months of GAHT, showing a 41 % reduction. **CBG levels** significantly increased from 46.5 mg/dl at baseline to 47.5 mg/dl after 3 months of GAHT, showing a 2.1 % increase.	**Baseline total serum cortisol** was not significantly different from baseline to 6 months of GAHT. Baseline free cortisol did not change significantly after 6 months of GAHT. Baseline salivary cortisol level changes from baseline to 6 months of GAHT were not reported. The **ratio of free cortisol to total cortisol** was not reported in the transmasculine group. **ACTH-stimulated total serum** cortisol +40 min after stimulation decreased from baseline (706.2 ± 107.6 nmol/L) to 6 months of GAHT (642.7 ± 148.9 nmol/L), showing a 9 % reduction. **ACTH-stimulated free cortisol** at +30 and + 40 min decreased from 48.5 ± 11 nmol/L and 41.4 ± 11 nmol/L at baseline and 44.67 ± 11 nmol/L and 37.2 nmol/L after 6 months of GAHT, showing reductions by 8 % and 10 %. **ACTH-stimulated salivary cortisol** levels at multiple timepoints decreased significantly from baseline (52.4 ± 24 nmol/L; 61.7 ± 26.2 nmol/L; 60.9 ± 35.6 nmol/L) to 6 months of GAHT (26.5 ± 8 nmol/L; 31.4 ± 8.5 nmol/L; 23.7 ± 9.4 nmol/L), resulting in reductions of 51 %, 49 % and 61 %.
**Results in transfeminine group**	**Serum cortisol levels** decreased from 607.42 nmol/l (243.95) at baseline to 347.79 nmol/l (149.72) after 12 months and 304.32 nmol/l (119.00) after 24 months of GAHT, showing decreases of 43 % and 50 %, respectively. **CBG levels** increased from 103.63 μg/ml at baseline to 363.54 μg/ml after 12 months of GAHT and 446.04 μg/ml after 24 months of GAHT, showing increases of 351 % and 430 %, respectively.	**Baseline serum cortisol** increased from 189.8 ± 43.5 μg/l at baseline to 228.7 ± 51.9 μg/dl after 3 months of GAHT, showing a 20 % increase. **CRH-stimulated cortisol** increased from 2970 ± 1257.5 μg/dl × min at baseline to 3354.1 ± 2341 μg/dl × min after 3 months of GAHT, showing a 13 % increase. **CBG levels** increased from 46.4 mg/dl at baseline to 49.6 mg/dl after three months of GAHT, resulting in a 6.9 % increase.	**Baseline total serum cortisol** increased from baseline (510 ± 152 nmol/L) to 6 months after GAHT (562.83 ± 151 nmol/L), resulting in a 10 % increase. The **ratio of free cortisol to total cortisol** decreased from 10.21 ± 7.1 % at baseline to 6.41 ± 1.13 % after 6 months of GAHT. **Baseline salivary cortisol** levels did not change after 6 months of GAHT. **ACTH-stimulated total and free serum** cortisol did not change between baseline an after 6 months of GAHT. **ACTH-stimulated salivary cortisol** levels at +20 and + 30 min decreased from baseline (55.1 ± 33.6 nmol/L and 56.8 ± 33.9 μg/dL) to 6 months of GAHT (29.8 ± 9.9 nmol/L and 37.2 ± 11.8 μg/dL), resulting in decreases of 54 % and 65 %.

Taken together, these studies by Mueller et al. (2006), Fuss et al. (2019) and Sofer et al. (2024) show that GAHT could affect HPA axis dynamics, but none of the studies examined diurnal cortisol concentrations, and only Sofer et al. (2024) reported free cortisol fractions. Thus far, studies on diurnal salivary cortisol in transgender people have not focused on GAHT, and have mostly studied social stressors. These studies indicate that transgender people are more likely to show lower diurnal cortisol slopes due to social stress (DuBois et al., 2017, 2024), indicating that transgender people might already have less advantageous cortisol slopes, but no study has examined the effects of GAHT on salivary diurnal cortisol slopes.

Examining diurnal salivary cortisol concentrations is more relevant for well-being compared to single time-point or stimulated serum cortisol levels for two reasons. Firstly, salivary cortisol is a better reflection of the amount of bioavailable cortisol compared to total cortisol measured in serum, since it shows how much cortisol is available to directly interact with its target receptors in various tissues and therefore affect physical and mental functioning. Secondly, measurement of the diurnal cortisol concentrations is more informative for well-being than single time-point measurements, since there are large individual differences in healthy cortisol serum levels, whereas the diurnal slope is an important proxy of healthy HPA axis functioning (Adam et al., 2017).

Therefore, this study aimed to examine changes in diurnal cortisol levels in saliva in transgender people starting GAHT in two ways. First, we examined existing sex differences in diurnal cortisol prior to starting GAHT, comparing participants assigned female and male at birth. Second, we examined in saliva the changes in diurnal cortisol after three months of feminizing and masculinizing GAHT.

## 2 Methods

### 2.1 Participants and setting

Data collection for this research was part of the RESTED study, in which we primarily aimed to examine changes in sleep and depression after 3 and 12 months of GAHT use. The RESTED study recruited participants from the Amsterdam UMC and University Medical Centre Groningen who were aged between 18 and 50 years old, had proficiency in Dutch language and who did not use any sleep medication. The medical ethical committee of the Amsterdam UMC (location VUmc) declared that the Medical Research Involving Human Subjects Act (WMO) did not apply to data collection for the RESTED study (study code 2019.353). Before inclusion in the study, all participants received written and oral information about the study, and all participants provided informed consent. Participants recruited from May 2021 to September 2023 from the Amsterdam UMC were invited to partake in the salivary cortisol collections during the study, and all saliva samples were collected between June 2021 and August 2022. Out of 54 study participants eligible for participation, 6 participants successfully took part in only the baseline saliva sampling, 1 participant successfully took part in only 3-month saliva sampling and 18 participants successfully took part in both the baseline and 3-month saliva sampling. We opted to only include participants who took part in both baseline and 3-month saliva sampling, resulting in a participation rate of 33 %.

#### 2.1.1 Demographic characteristics

Demographic characteristics were obtained from clinical records (i.e., age, alcohol, smoking and psychotropic medication) and from survey questionnaires (i.e., perceived stress score). Survey questionnaires were sent to participants at every measurement timepoint in the study using Castor EDC, and perceived stress was measured using the 10-item Perceived Stress Scale (PSS; Cohen et al., 1983). The PSS is scored on a Likert scale from 0 to 4, resulting in a score range from 0 to 40, with a higher score indicating higher perceived stress levels. Study results on changes in depression (Morssinkhof et al., 2024), sleep (Morssinkhof et al., 2023) and perceived stress (Schipper et al., 2025) after starting GAHT are reported elsewhere.

### 2.2 Gender-affirming hormone care

#### 2.2.1 GAHT

All participants received gender-affirming care in line with the Standards of Care of the World Professional Association for Transgender Health (WPATH; Coleman et al., 2022). Participants were grouped in either the transmasculine (TM) or transfeminine (TF) group, based on the form of GAHT they received, which was in turn determined by their sex assigned at birth. Transmasculine (TM) participants (all assigned female at birth, starting masculinizing GAHT) started using testosterone gel (daily dose of 40.5 mg) or testosterone esters (250 mg every 3 weeks). Several transmasculine participants used progestins to suppress menstruation (lynestrenol, 5 mg; levonorgestrel IUD, 52 mg; medroxyprogesterone, 150 mg), and two transmasculine participants still used progestins (medroxyprogesterone, 150 mg) after three months of GAHT. Transfeminine (TF) participants (all assigned male at birth, starting feminizing GAHT) started estradiol tablets (estradiol valerate, 2 mg twice daily), estradiol gel (0.06 % 1.5 mg daily) or estradiol patches (100 mcg per 24 h, twice a week) as well as anti-androgens, either short-acting gonadotropin-releasing hormone analogues (GnRH analogues; triptorelin 3.75 mg per 4 weeks, or leuproreline, 3.75 mg per 4 weeks) or long-acting GnRH analogues (triptorelin 11.25 mg per 12 weeks). Treatment specifications of the participants are displayed in Table 2.

**Table 2:** Clinical and demographic baseline characteristics of study participants.

		Transmasculine (TM) group	Transfeminine (TF) group
Sample size in total cohort		11	7
Age a	*Mean (SD)*	21.3 (2.5)	28.0 (5.6)
Alcohol (consumptions per week)	*Mean (SD)*	0.4 (0.6)	0.8 (1.9)
Smoking (n, %)	*Never*	8 (72 %)	7 (100 %)
	*Previous*	2 (18 %)	0 (0 %)
	*Current*	1 (9 %)	0 (0 %)
Psychotropic medication use at baseline (n, %)	*Antidepressants*	2 (18 %)	1 (14.2 %)
	*Anxiolytics*	0 (0 %)	0 (0 %)
	*Stimulants*	2 (18 %)	0 (0 %)
	*Antipsychotics*	0 (0 %)	0 (0 %)
Perceived Stress Score at baseline	*Mean (SD)*	12.9 (6.8)	12.6 (8.8)
Cycle regulation use at baseline (n, %) b		4 (36 %)	–
Form of testosterone prescribed at start of GAHT (n, %)	*Testosterone gel*	8 (73 %)	–
	*Short-acting injections*	3 (27 %)	–
	*Long-acting injections*	0 (0 %)	–
Form of estrogen prescribed at start of GAHT (n, %)	*Estradiol tablets*	–	6 (86 %)
	*Estradiol patches*	–	1 (14 %)
Form of anti-androgens prescribed at start of GAHT (n, %)	*GnRH analogues*	–	7 (100 %)

a The transmasculine group was significantly younger than the transfeminine group (p = 0.018).

b 2 participants used intramuscular medroxyprogesterone injections, 1 used lynestrenol tablets, 1 had a medroxyprogesterone implant.

### 2.3 Laboratory analysis

#### 2.3.1 Sex hormone methods

Blood withdrawal was performed at clinic intake (i.e., before starting GAHT) and at a clinic visit after 3 months of GAHT. After centrifugation, serum was stored in the freezer until analysis took place within 2 weeks time. Serum testosterone and estradiol levels were measured using in-house developed liquid chromatography-tandem mass spectrometry (LC-MS/MS) methods which were described earlier (Büttler et al., 2016; Fanelli et al., 2024; Verdonk et al., 2019). The testosterone method had a lower limit of quantitation of 0.1 nmol/L and an inter-assay coefficient of variation of 4 % at a concentration of 24 nmol/L, and the estradiol method had a lower limit of quantitation of 21 pmol/L and an inter-assay coefficient of variation of 5.1 % at a concentration of 185 pmol/L. Sex hormone binding globulin (SHBG) was determined using a non-competitive sandwich immunoassay (Alinty, Abbott Diagnostics, Chicago, USA), with a lower limit of quantitation of 4.5 nmol/L and an inter-assay coefficient of variation of 4.7 % at a concentration of 51 nmol/L.

#### 2.3.2 Saliva sample collection

Saliva sampling was performed using Salivettes (Sarstedt, Numbrecht, Germany). Before the saliva sampling, participants were instructed to rinse their mouth and abstain from eating for at least 10 min before taking the sample. Participants received a saliva sampling kit with four color- and number-coded saliva tubes accompanied by a user guide and sampling diary, in which they could track the sampling times. Participants were instructed to place the swab in their mouth and lightly chew it for at least 90 s, or until saturated, and then put the swab into the Salivette tube. Participants were instructed to conduct saliva assessments at four fixed timepoints during their day: 30 min (T1), 5.5 h (T2) and 10.5 (T3) hours after awakening as well as 30 min before going to bed (T4), and they were instructed to note the exact time of sampling using paper diaries. One participant did not hand in the paper diaries with sampling times, and their sample times were imputed based on the instructed times for T1, T2 and T3 (i.e., 30 min, 5.5 h and 10.5 h after awakening) and based on the cohort mean for T4. Collected samples were kept at home at 4° Celcius (i.e., domestic refrigerator temperature) until they were returned to the research team for analysis. After the samples were returned, they were centrifuged, pipetted and frozen at −80 °C until analysis.

#### 2.3.3 Laboratory analysis

Salivary cortisol concentrations were measured in the Endocrine Laboratory of the Amsterdam UMC using an isotope dilution LC–MS/MS method. In short, internal standard (13C3-labeled cortisol, Sigma Aldrich) was added to the samples. Samples were extracted by supported liquid extraction (Biotage) and analyzed on an LC-MS/MS (Xevo TQ-S Micro LC-MS-MS System, 10.13039/100024208Waters Corporation). The lower limit of quantitation (LLOQ) was 0.5 nmol/L. The intra-assay variation was 5 % and 3 % at cortisol concentrations of 2 and 15 nmol/L. The interassay variation was 8.2 % at a level of 1.9 nmol/L, 5.6 % at a level of 9 nmol/l and 5.3 % at a level of 44 nmol/L. Samples below the LLOQ were set at half the value of the LLOQ (i.e., 0.25 nmol/L).

### 2.4 Analysis

#### 2.4.1 Calculation of outcomes

To assess total exposure to cortisol during the day, we calculated the mean cortisol level per time point (i.e., baseline and 3 months of GAHT) as well as the area under the curve (AUC) for the daytime slope per time point, which was calculated as the Area Under the Curve with respect to ground (AUCg; Pruessner et al., 2003). To assess cortisol dynamics across the day, we calculated the difference between the wake- and bedtime cortisol levels by subtracting the levels at T4 from the levels at T1, and we calculated the diurnal linear and quadratic slopes (i.e., estimated change in cortisol per hour) using hierarchical linear models.

#### 2.4.2 Statistical analyses

The statistical analyses were all based on frequentist statistics. They were conducted in Rstudio, using the “lme4” package (Bates et al., 2015). To examine baseline differences between the TM and TF groups in absolute cortisol levels per measurement (i.e., T1, T2, T3 and T4), daily average cortisol levels, AUCg's and wake-bedtime slopes between the groups, we used unpaired t-tests. To examine baseline group differences in the diurnal slopes of cortisol over the course of the day, we created a model accounting for the linear and quadratic slopes over the day. This model consisted of the absolute cortisol levels as the outcome variable, the time since awakening as the predictor with an interaction term for group (i.e., TM or TF group), the squared time since awakening as a second predictor with an interaction term for group and a random intercept per participant.

To examine changes in outcomes after starting GAHT, we stratified analyses per group, analyzing the TM and TF group separately. We used paired t-tests to examine changes in absolute cortisol levels per measurement (i.e., T1, T2, T3 and T4), daily average cortisol levels, AUCg's and wake-bedtime slopes. To examine changes in the linear and quadratic diurnal slopes after GAHT, we used a diurnal cortisol slope model again, with the absolute cortisol levels as the outcome variable, the time since awakening as predictor with an interaction term for measurement (i.e., baseline or 3-month measurement), the squared time since awakening as a second predictor with an interaction term for measurement, as well as a random intercept per participant.

We conducted a sensitivity analysis on the absolute cortisol levels, mean cortisol levels, AUCg's and wake to bedtime-slopes using the Bayesian *t*-test framework (see also Jeffreys, 1961; Rouder et al., 2009), using JASP software (JASP Team, 2024). Our a priori hypothesis for our first aim (i.e., H_A_) was that TM participants would have higher cortisol levels and steeper diurnal salivary cortisol slopes than TF participants at baseline, i.e. a one-sided hypothesis assuming a positive value of δ for the TF vs. TM group for our hypotheses for absolute cortisol levels, mean cortisol and AUCg, and a negative value of δ for the wake to bedtime-slope. Our null hypothesis was that there would be no difference between the groups, assuming δ = 0. For analyses examining this first hypothesis, we used the Bayesian analysis versions of independent t-tests (Rouder et al., 2009) with δ set at the default level, i.e. a Cauchy prior distribution with r = 1/√2, but limited to only allow positive values of δ for absolute cortisol levels, mean cortisol and AUCg and only negative levels for the wake to bedtime-slope. Our hypotheses for the second research aim (i.e., H_A_) were that after starting GAHT, TM participants would show reductions in absolute cortisol levels and a less steep diurnal slope, whereas TF participants would show increases in absolute cortisol levels and a steeper diurnal slope. To examine these hypotheses, we used Bayesian analysis versions of one-sided paired t-tests with the δ set at the default level, i.e. a Cauchy prior distribution. For the TM group, we limited the prior distribution to only negative values of δ for the absolute cortisol levels, mean cortisol level and AUCg and to only positive values of δ for the wake to bedtime-slope. For the TF group, we limited the prior distribution to only positive values of δ for the absolute cortisol levels, mean cortisol level and AUCg and to only negative values of δ for the wake to bedtime-slope.

Please note that, due to the explorative nature of this study and the conceptual and statistical overlap between the outcomes in this study, we did not do multiple testing correction in this study. This means that the results should be interpreted with caution, as the Type I error rate might be higher than the nominal 0.05 alpha level. For replication and transparency purposes, the code for all R analyses and the JASP analysis results, including input options and plots, are available in Supplementary materials and via osf.io/8dnjp/.

## 3 Results

### 3.1 Demographic and clinical characteristics

A total of 18 participants, of whom 11 transmasculine and 7 transfeminine participants, collected saliva for cortisol analysis. All participants provided four cortisol samples at baseline and after three months of GAHT. As displayed in Table 2, the transmasculine group was significantly younger than the transfeminine group (21 years old vs. 28 years old). Comparison of the PSS scores shows no significant differences between the TM and TF group at baseline (p = 0.93) and no significant changes after 3 months of GAHT in the TM (mean = 12.3, SD = 6.9, p = 0.82) or TF (mean = 11.7, SD = 8.8, p = 0.42) group.

### 3.2 Serum sex hormone concentration

Mean testosterone levels in the transmasculine group were 1.1 nmol/L (SD 0.5) at baseline and 19.4 nmol/L (SD 14.7) after three months of GAHT, and mean sex hormone binding globulin (SHBG) levels were 37 nmol/L (SD 14) at baseline and 28 nmol/L (SD 11) after three months of GAHT. Mean testosterone levels in the transfeminine group were 14.8 nmol/L (SD 6.7) at baseline and 0.53 nmol/L (SD 0.2) after three months of GAHT, mean estradiol levels in the transfeminine group were 75 pmol/L (SD 23) at baseline and 351 pmol/L (SD 80) after three months of GAHT, and mean SHBG levels were 40 nmol/L (SD 19) at baseline and 84 nmol/L (SD 30) after three months of GAHT.

### 3.3 Salivary cortisol concentrations

**Fig. 1:**
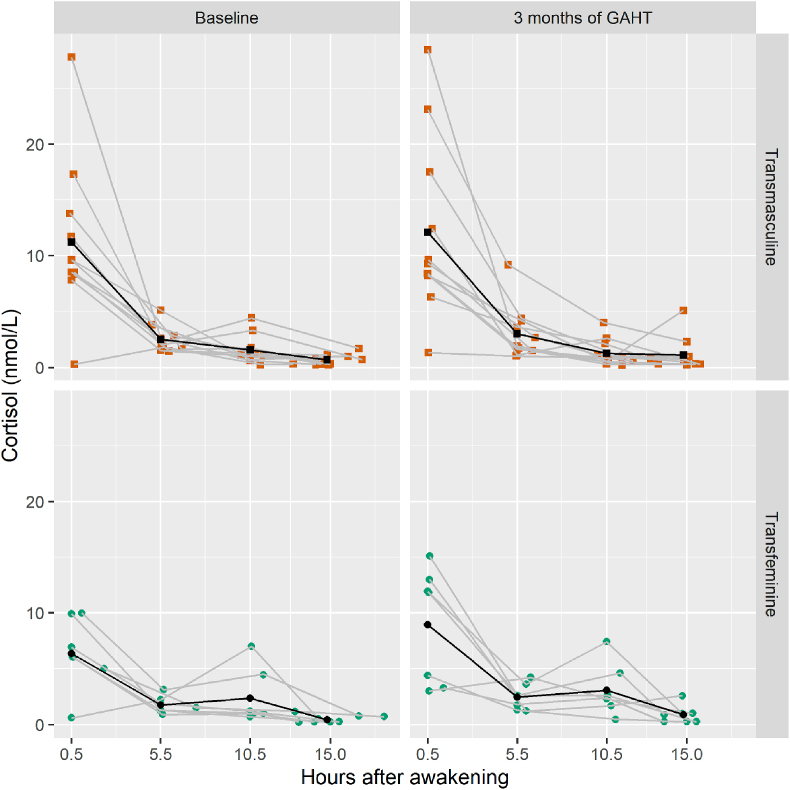
**Salivary cortisol concentrations ordered by time after awakening, plotted per participant and overall mean.** The black line and dots show the group means per sample, and colored dots display the actual time since awakening, and the grey lines connect each participant's measures.

At baseline, we find that the transfeminine group shows a significantly less steep linear diurnal slope compared to the transmasculine group (TF vs. TM: +1.1 nmol/L × hour, 95 % CI: 0.2 to 2.1, p = 0.02) but we find no significant group differences in the quadratic diurnal slope. We also find that the transfeminine group and transmasculine group show no significant differences in absolute cortisol levels, daily average cortisol or wake to bedtime-slopes (Table 3, Fig. 1).

**Table 3:** Salivary cortisol concentrations before and after three months of GAHT.

	Transmasculine group			Transfeminine group		
	n = 11			n = 7		
	Baseline	Three months of GAHT	Three months vs. baseline	Baseline	Three months of GAHT	Three months vs. baseline
	*Absolute cortisol levels (hours after awakening)*					
T1: + 0.5 h (nmol/L)	11.2 (6.9)	12.1 (7.9)	p = 0.82	6.3 (3.2)	8.9 (5.2)	p = 0.25
T2:+ 5.5 h (nmol/L)	2.5 (1.1)	3.0 (2.4)	P = 0.59	1.7 (0.7)	2.5 (1.1)	P = 0.06
T3:+ 10.5 h (nmol/L)	1.5 (1.2)	1.3 (1.2)	P = 0.63	2.4 (2.4)	3.1 (2.3)	p = 0.52
T4: 0.5 h before bedtime (nmol/L)	0.7 (0.5)	1.1 (1.5)	P = 0.12	0.4 (0.2)	0.9 (0.8)	P = 0.40
	*Total daily cortisol*					
Daily Average Cortisol (nmol/L)	4.0 (2.1)	4.4 (2.7)	P = 0.77	2.7 (0.9)	3.8 (1.8)	P = 0.15
Area Under the Curve (nmol/L^a^ hours)	50.5 (25.1)	50.5 (31.4)	p = 0.85	36.8 (14.3)	50.6 (23.3)	P = 0.13
	*Cortisol slope*					
Wake-Bedtime slope (nmol/L)	−10.6 (6.6)	−11.0 (6.8)	p = 0.90	−5.9 (3.2)	−8.0 (4.8)	p = 0.30
Linear diurnal slope^a^ (nmol/L^a^hours)	−1.9 (−2.5 to −1.3), p < 0.001	−2.2 (−3.0. to −1.4), p < 0.001	−0.2 (−0.8 to 1.3), p = 0.67	−0.8 (−1.4 to −0.2), p = 0.02	−1.3 (−1.9 to −0.6), p < 0.001	0.5 (−0.4 to 1.4), p = 0.29
Quadratic diurnal slope^a^ (nmol/L^a^ hours^2^)	0.08 (0.04–0.12) P = 0.001	0.09 (0.04–0.14) P < 0.001	0.01 (−0.8 to 0.05), p = 0.70	0.03 (−0.01 to 0.06), p = 0.19	0.05 (0.01–0.09), p = 0.03	0.02 (−0.08 to 0.03), p = 0.41

a Analyzed in one model with both linear and quadratic slopes. The cells in the Baseline and Three months of GAHT columns display the means and standard deviations of the outcomes, except for the cells displaying the linear and diurnal slope, which display the estimated slope. Cells in the Three months vs. Baseline columns display the results of statistical analyses comparing the two time points.

Analyses examining changes in cortisol levels after starting GAHT in the transmasculine group do not find any significant changes in absolute cortisol levels, daily cortisol levels, area under the curve or linear or quadratic diurnal cortisol slopes after three months of GAHT (Table 3, Fig. 1). In the transfeminine group, analyses also show no significant changes in absolute cortisol levels, daily mean cortisol level during the day, wake to bedtime-slope, area under the curve or in linear or quadratic diurnal slopes (Table 3, Fig. 1).

### 3.4 Sensitivity: Bayesian statistics

To assess the likelihood of null results in our sample, we conducted Bayesian analyses as sensitivity analyses. As shown in Supplementary Table 1, Bayes factors from the baseline analyses (i.e., group comparisons) indicate that there is only anecdotal evidence for group differences all outcomes at baseline, meaning we cannot draw any meaningful conclusions about either the H_0_ or H_A._ There is one exception for sample 3, where analyses indicate moderate evidence for the null hypothesis (i.e., no difference between the TM and TF group at baseline; BF = 0.25), indicating that it is four times more likely that the H_0_ is true compared to the H_A_. Please note that we did not analyze the diurnal slope differences between the groups in the Bayesian analyses, and we therefore do not assess whether we replicate this group difference in this section.

Analyses examining changes after starting GAHT indicate that in the TM group, there is moderate evidence for the H_0_ (i.e., no changes after 3 months of GAHT) for sample 1, 2, 4, the average daily cortisol, the AUCg and the wake to bedtime-slope. Bayes factors are between 0.18 and 0.27, meaning it is 3.7–5.5 times more likely that the H_0_ is true compared to the H_A_ for these outcomes. In the TF group, our analysis indicates moderate evidence for our H_A_ (i.e., increases after 3 months of GAHT) for sample 2: the Bayes factor of 3.24 indicates that it is three times more likely that the H_A_ is true compared to the H_0_. Furthermore, analyses indicate moderate evidence for the null hypothesis (i.e., no changes after 3 months of GAHT) for the average daily cortisol and the AUCg, with Bayes factors of 0.17 for both outcomes, indicating that it is 5.9 times more likely that the H_0_ is true compared to the H_A_ for these outcomes. For all other outcomes, evidence for either the H_0_ or H_A_ is anecdotal, meaning the results cannot meaningfully provide support for either the H_0_ or H_A_. Full results, including error percentages and robustness analyses, are reported in supplementary materials.

## 4 Discussion

In this study, we examined the effects of three months of GAHT on diurnal salivary cortisol levels. Our findings at baseline replicate some of the aforementioned sex differences, showing a steeper diurnal cortisol slope in transmasculine participants, who were assigned female at birth, compared to the transfeminine participants, who were assigned male at birth, although we find no significant differences in absolute or mean salivary cortisol levels. The observed steeper diurnal salivary cortisol slope is line with previous research in cisgender adults (Larsson et al., 2009), confirming our hypothesis of sex differences in diurnal cortisol levels.

Comparisons of diurnal salivary cortisol samples between baseline and 3 months of GAHT show no significant changes in either the transmasculine or transfeminine participant groups. Bayesian sensitivity analyses indicate that for most outcomes in the transmasculine group, there is more likely no difference after 3 months of GAHT compared to baseline, although these analyses find mixed results across the outcomes in the transfeminine group. Our results are somewhat in line with salivary cortisol levels reported by Sofer et al. (2024) in their transfeminine group, who find no changes in salivary cortisol levels, but they are in contrast with what we expected based on studies examining serum cortisol and CRH- and ACTH-stimulated cortisol levels in trans people who started GAHT.

Our results mostly show no significant changes in diurnal cortisol dynamics after 3 months of feminizing GAHT, and a high likelihood of no changes in diurnal cortisol dynamics in after 3 months of masculinizing GAHT. These results are different from our hypotheses, but they paint a possibly reassuring picture if we consider the clinical and real-life relevance: our results show that participants preserve their endogenous diurnal cortisol slope, with most likely no adverse effects of short-term GAHT use on salivary cortisol. This is especially reassuring considering that transgender people are more likely than cisgender people to be exposed to external stressors, including discrimination and minority stress, which have been shown to adversely affect diurnal cortisol levels. However, we want to stress that the overall sample size of this study is quite low, the duration of our follow-up is short, and further replication in larger samples and with longer follow-up is needed.

**Fig. 2:**
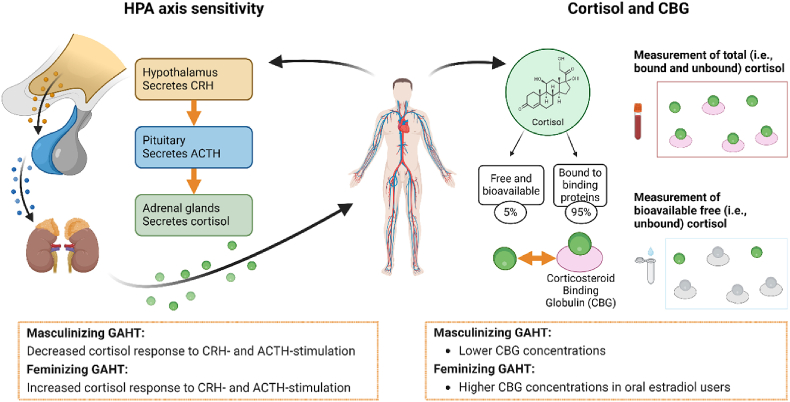
Illustration of the factors affecting bioavailable cortisol concentrations and corresponding changes after starting GAHT. Based on our results as well as results of Mueller et al. (2006), Fuss et al. (2019), Sofer et al. (2024) and Stangl et al. (2025).

Although there is an apparent gap between our findings and previous studies, there are numerous ways in which GAHT could affect the HPA axis which would not be reflected in salivary diurnal cortisol levels. We will reflect on two of these mechanisms and their implications, and both mechanisms are also illustrated in Fig. 2.

Firstly, the aforementioned studies show indications of GAHT changing HPA axis responsivity, finding increasing or decreasing cortisol production in response to exogenous administration of CRH or ACTH. In rodents, estradiol affects HPA axis responsivity via binding to estrogen receptors in the hypothalamus (Isgor et al., 2003). Specifically, estradiol can activate estrogen receptor β in hypothalamic CRH neurons (Laflamme et al., 1998; Mitra et al., 2003), and hypothalamic estrogen receptor-α also modifies HPA axis responsivity (Weiser and Handa, 2009). Furthermore, estradiol could decrease CRH effectivity through increases in the level of CRH-binding proteins (van de Stolpe et al., 2004). In this context, it is important to note that stimulation of the HPA axis through exogenous CRH or ACTH cannot be generalized to endogenous HPA axis function.

Secondly, it is possible that GAHT changes both cortisol and CBG concentrations, as seen in Sofer et al. (2024) and (Stangl et al., 2025). In that case, the increased cortisol secretion cortisol is then “buffered” by an increase in binding proteins for cortisol. Based on studies on the oral contraceptive pill, which show that CBG concentrations increase within four weeks after participants start OC use (van der Vange et al., 1990; Wiegratz et al., 2003), we assume that CBG concentrations also change within 3 months after participants start GAHT. If this were the case, our results would indicate that an increase in total cortisol after starting feminizing GAHT could be compensated by an increase in CBG levels, or a decrease in total cortisol after starting masculinizing GAHT could be compensated by a decrease in CBG levels, meaning any effects of GAHT use would not show up in changes in diurnal free cortisol.

It is important to note that our results only reflect the unstimulated diurnal slope without accounting for the role of stress. The HPA axis responsivity to stress can affect the magnitude and duration of stress-driven cortisol increases. CBG plays an important role in protecting and transporting cortisol (Henley et al., 2016), and CBG could moderate availability of cortisol in stress reactions, with one study showing that participants with higher CBG levels showed stronger cortisol increases in response to stress (Kumsta et al., 2007). Therefore, future studies should also assess the effect of GAHT on CBG and cortisol in stress reactions.

Our study has numerous strengths to consider. This study was the first to assess diurnal salivary cortisol in transgender people who started GAHT. We examined the fraction of free unbound cortisol meaning that the cortisol concentrations we measured are more relevant for biobehavioral mechanisms compared to total or bound cortisol concentrations. The naturalistic setup, asking participants for ambulatory saliva samples at times relevant to their own waking and bedtimes, strengthens the external validity of the study. All samples were analyzed using LC-MS/MS, which is a more reliable method of assessing cortisol levels compared to immunoassays (Hillebrand et al., 2021), which have often been used in past work on this topic. The prospective setup of the study, with repeated measurements in all participants before GAHT and after three months of GAHT, strengthens the extent to which we can assume or rule out a causal role of GAHT in changes in salivary cortisol.

This study, however, also has limitations which should be taken into account. Firstly, the sample size of our study is small, with seven transfeminine participants and eleven transmasculine participants. This is partially due to the high burden of the sampling protocol (i.e., taking four samples at set times during the day, keeping these samples in a fridge after sampling) and due to low willingness to participate and loss to follow-up for this subpart of the study, as illustrated by the 33 % successful participation rate. Since this was the first study to examine salivary diurnal cortisol, we could not do an a priori power analysis, so we could not be certain whether we could achieve adequate power to detect any existing effects of GAHT before our analyses. However, we conducted post-hoc sensitivity analyses using Bayesian methods, which give further information about the likelihood of null results in our sample. Future studies aiming to examine the diurnal slope of salivary cortisol should power their study for a very small to medium effect size. Considering that very large sample sizes this large might not be realistic, the addition of repeated measures (i.e., measuring every day for a longer period of time) or the use of Bayesian statistics could be helpful to bolster power and further insight in future studies.

Secondly, although the use of saliva samples to measure cortisol is easier and less invasive than serum measurements, saliva samples are also prone to user errors, including eating or drinking before sampling, taking the sample at the wrong time, as well as to blood contamination (Brescia et al., 2016; Kudielka et al., 2003). Participants also tracked the times of the cortisol samples on paper, which is less reliable than the use of digital tracking of sample timing compliance (Jacobs et al., 2005; Moeller et al., 2014).

Thirdly, as shown in Table 2, numerous transmasculine participants were using progestin-only cycle regulation. Exogenous progestins are known to affect salivary cortisol levels, although studies show that especially the CAR and cortisol responses to stress could be most strongly affected by progestins (Herrera et al., 2019; Høgsted et al., 2021). The effects of progestin-only contraceptives on HPA axis outcomes are relatively unknown and needs further study (Özcan et al., 2024).

Fourth, diurnal cortisol is also strongly affected by psychosocial stress. Transgender people experience a significantly higher amount of social stress due to stigma and discrimination compared to cisgender people, and studies thus far show that high levels of perceived stress and stressors related to transition (i.e., not being perceived as one's gender identity, bathroom stress, stress about coming out) were associated with higher awakening cortisol levels (Colizzi et al., 2013; DuBois et al., 2017). As shown in Table 2, the current cohort shows relatively low levels of perceived stress at baseline and perceived stress does not significantly change after 3 months of GAHT in either group. It is nonetheless possible that there are differences in transition-related stressors between the transmasculine group and the transfeminine group (Poquiz et al., 2021). Although some, but not all studies find reductions in perceived stress after 12 months GAHT (Ceolin et al., 2024; Colizzi et al., 2013; Schipper et al., 2025), thus far there is no research on the short-term effects of GAHT on minority- or transition-related stress. Future studies should incorporate both social (e.g., minority stress) and endocrine (e.g., GAHT) measures into studies on cortisol dynamics in transgender people.

Lastly, this study is also limited by the number of cortisol samples, the follow-up duration and the lack of a control group. We collected saliva samples at four time points, but we did not collect sufficient samples to phenotype the CAR, for which we would have needed an additional sample collected immediately after awakening and at least one sample 45 or 60 min after awakening (Nasser et al., 2023). Some studies find that the CAR is a more sensitive measure of HPA axis functioning, and future studies could build upon this work by measuring the full CAR. Furthermore, the length of follow-up in this study is relatively short, since we only measure changes after 3 months of GAHT, while in clinical practice participants are expected to possibly use GAHT for decades. It is possible that diurnal cortisol changes develop later during the hormone treatment. Additionally, we did not include a cisgender control group, which could have been helpful to provide context to the sex differences in the baseline findings. Altogether, further research should examine the CAR, longer term effects of GAHT and possibly include a cisgender control group if relevant.

In summary, our findings replicate previously identified sex differences in diurnal salivary cortisol levels, mainly a steeper diurnal slope in those assigned female at birth, but we find no indications for meaningful changes in salivary diurnal cortisol after 3 months of GAHT. It is important to note that this study has a small sample size, a short follow-up duration, our results are limited to diurnal salivary cortisol levels, and studies on cortisol increases in response to (social) stress after GAHT are still lacking. Based on the possible effects of GAHT on HPA axis responsivity and concentrations of CBG, future studies on GAHT and cortisol should focus on the interaction of CBG and cortisol as well as on examining the effects of GAHT cortisol responses to stress.

## CRediT authorship contribution statement

**Margot W.L. Morssinkhof:** Writing – original draft, Visualization, Investigation, Formal analysis, Data curation. **David Matthew Doyle:** Writing – review & editing, Supervision, Funding acquisition, Formal analysis. **Ysbrand D. van der Werf:** Writing – review & editing, Investigation. **Martin den Heijer:** Writing – review & editing, Investigation. **Annemieke Heijboer:** Writing – review & editing, Validation, Resources. **Birit F.P. Broekman:** Writing – review & editing, Supervision, Investigation, Funding acquisition. **Dirk Jan Stenvers:** Writing – review & editing, Investigation, Conceptualization.

## Declaration of competing interest

Birit F.P. Broekman reports financial support was provided by 10.13039/501100001826Netherlands Organisation for Health Research and Development. David Matthew Doyle reports financial support was provided by 10.13039/501100000780European Union. All other authors declare that they have no known competing financial interests or personal relationships that could have appeared to influence the work reported in this paper.

## Data Availability

Data will be made available on request.
