# Supplementary material for: Effects of gender-affirming hormones on diurnal cortisol concentrations: A prospective study
Source: Neurobiol Stress. 2025 Jun 18;37:100741. doi: 10.1016/j.ynstr.2025.100741 (PMC12264607; doi:10.1016/j.ynstr.2025.100741)
Supplement: Multimedia component 1 [file mmc1.docx]

# Results from Bayesian analyses

## Summary of all results

| **Supplementary Table 1. Results of sensitivity analyses using Bayesian analyses.** | | | |
| --- | --- | --- | --- |
| **Outcome** | **Hypothesis** | | |
|  | Baseline: H_A_ = TM > TF | In TM group: H_A_ = Baseline > 3MO | In TF group: H_A_ = Baseline < 3MO |
| **T1:  + 0.5 hours (nmol/L)** | BF_TM>TF_: 2.01  Error < 0.001% | BF_Base>3MO_: 0.25  Error < 0.001% | BF_Base<3MO_: 1.11  Error < 0.001% |
| **T2:**  **+ 5.5 hours (nmol/L)** | BF_TM>TF_: 1.69  Error < 0.001% | BF_Base>3MO_: 0.21  Error < 0.001% | BF_Base<3MO_: 3.24  Error < 0.001% |
| **T3:**  **+ 10.5 hours (nmol/L)** | BF_TM>TF_: 0.25  Error < 0.001% | BF_Base>3MO_: 0.44  Error < 0.001% | BF_Base<3MO_: 0.62  Error < 0.001% |
| **T4:  -0.5 hours before bedtime (nmol/L)** | BF_TM>TF_: 1.06  Error = ~ 0.016 | BF_Base>3MO_: 0.18  Error < 0.001% | BF_Base<3MO_: 1.91  Error < 0.001% |
| **Daily Average Cortisol (nmol/L)** | BF_TM>TF_: 1.50  Error < 0.001% | BF_Base>3MO_: 0.24  Error < 0.001% | BF_Base<3MO_: 0.17  Error < 0.001% |
| **Area Under the Curve (nmol/L * hours)** | BF_TM>TF_: 1.26  Error = ~  0.009% | BF_Base>3MO_: 0.26  Error < 0.001% | BF_Base<3MO_: 0.17  Error < 0.001% |
|  | Baseline: H_A_ = TM < TF | In TM group: H_A_ = Baseline < 3MO | In TF group: H_A_ = Baseline < 3MO |
| **Wake-Bedtime slope (nmol/L)** | BF_TM<TF_: 1.99  Error < 0.001% | BF_Base<3MO_: 0.27  Error < 0.001% | BF_Base>3MO_: 0.96  Error = ~ 0.019 |
| Bayes factors (BFs) are continuous outcomes indicating the likelihood of one hypothesis over another. Bayes factors of > 3 or < 0.33 are considered moderate evidence for one hypothesis over another, although the Bayes factor should not be interpreted based on these cutoffs alone. | | | |

## Hypothesis 1:

**At baseline, the transmasculine group has higher cortisol levels and a steeper diurnal slope than the transfeminine group**

Method: One-sided t-test (H_TM>TF_) for all outcomes, except the T4-T1 slope, which is (H_TM<TF_)

### Sample 1

| *Bayesian Independent Samples T-Test* | | | | | |
| --- | --- | --- | --- | --- | --- |
|  | | BF₊₀ | | error % | |
| cortisol.nmol.L |  | 2.009 |  | ~ 9.506×10^-6^ |  |
|  | | | | | |
| *Note.*  For all tests, the alternative hypothesis specifies that the location of group *TM* is greater than the location of group *TF* . | | | | | |

**cortisol.nmol.L**

**Prior and Posterior**


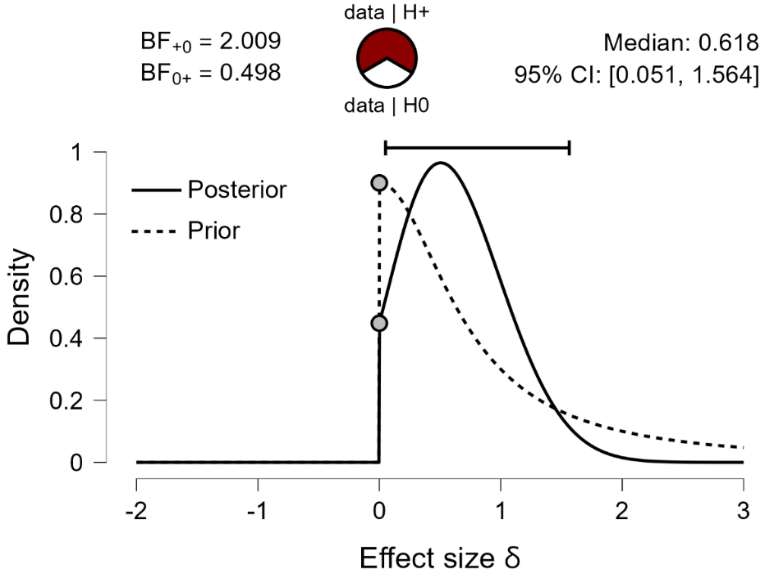


**Bayes Factor Robustness Check**


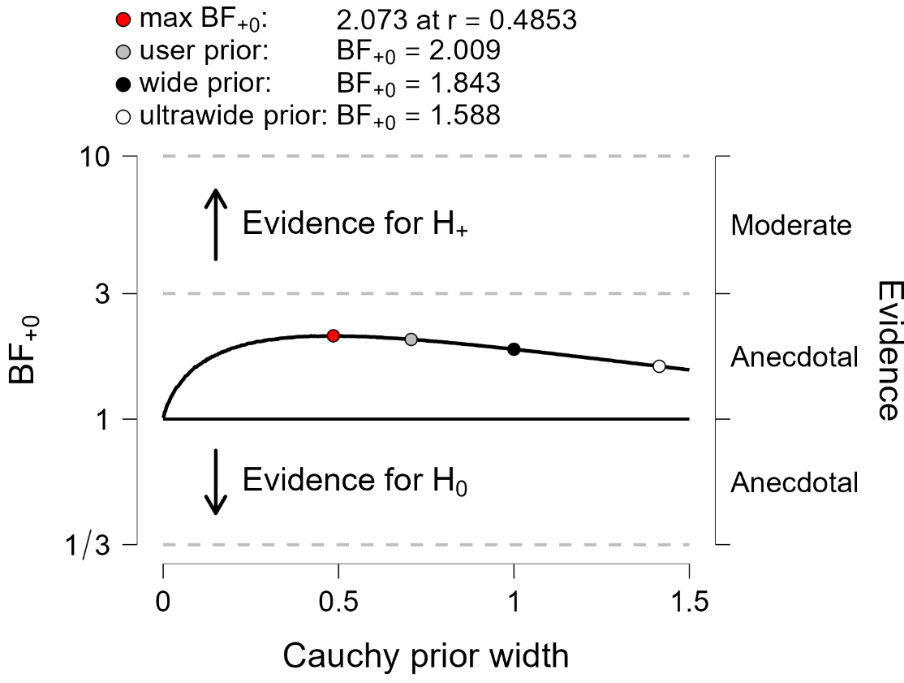


| *Descriptives* | | | | | | | | | | | | | | | | | |
| --- | --- | --- | --- | --- | --- | --- | --- | --- | --- | --- | --- | --- | --- | --- | --- | --- | --- |
|  | | | | | | | | | | | | | | 95% Credible Interval | | | |
|  | | Group | | N | | Mean | | SD | | SE | | Coefficient of variation | | Lower | | Upper | |
| cortisol.nmol.L |  | TM |  | 11 |  | 11.195 |  | 6.924 |  | 2.088 |  | 0.618 |  | 6.544 |  | 15.847 |  |
|  |  | TF |  | 7 |  | 6.343 |  | 3.198 |  | 1.209 |  | 0.504 |  | 3.385 |  | 9.300 |  |
|  | | | | | | | | | | | | | | | | | |

### Sample 2

| *Bayesian Independent Samples T-Test* | | | | | |
| --- | --- | --- | --- | --- | --- |
|  | | BF₊₀ | | error % | |
| cortisol.nmol.L |  | 1.686 |  | ~ 8.477×10^-6^ |  |
|  | | | | | |
| *Note.*  For all tests, the alternative hypothesis specifies that the location of group *TM* is greater than the location of group *TF* . | | | | | |

**cortisol.nmol.L**

**Prior and Posterior**


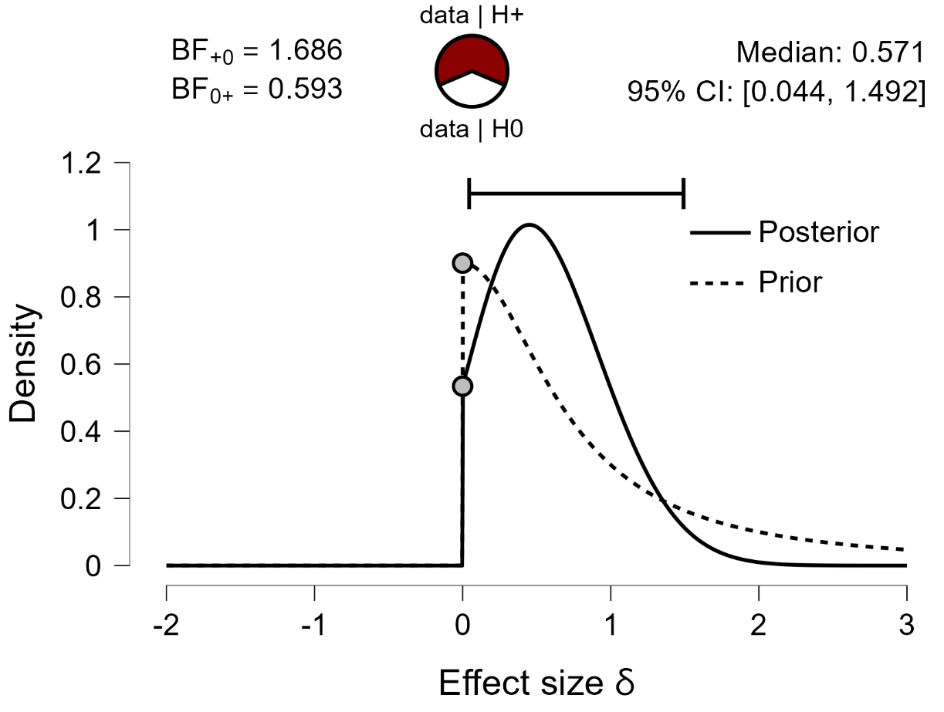


**Bayes Factor Robustness Check**


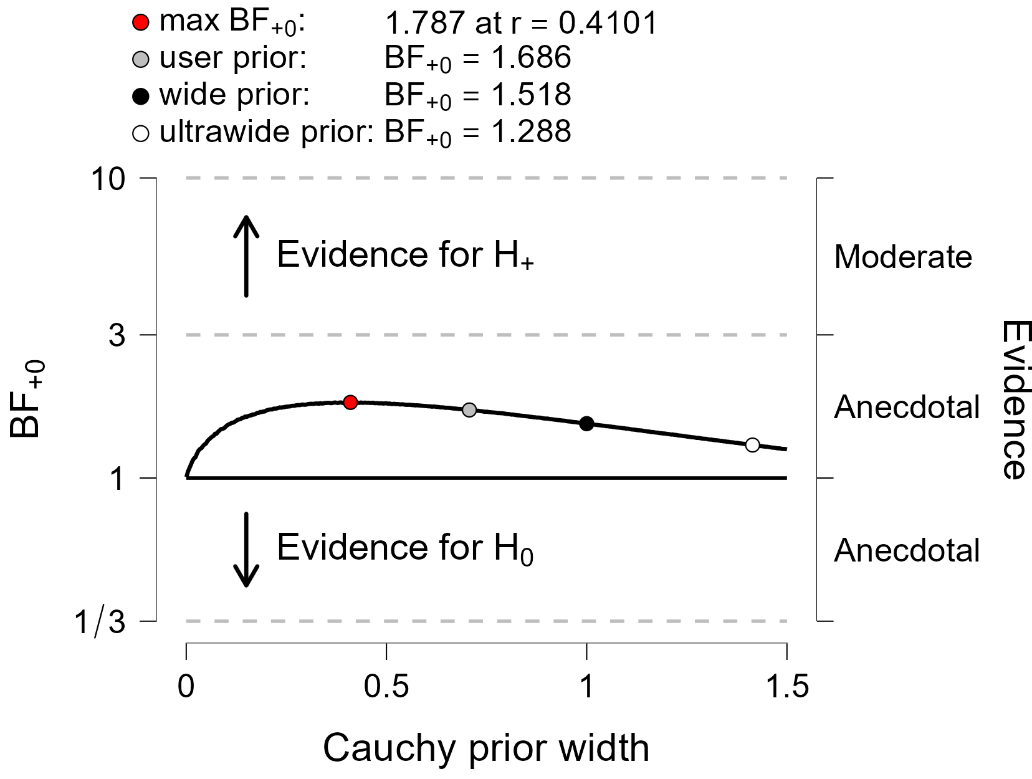


| *Descriptives* | | | | | | | | | | | | | | | | | |
| --- | --- | --- | --- | --- | --- | --- | --- | --- | --- | --- | --- | --- | --- | --- | --- | --- | --- |
|  | | | | | | | | | | | | | | 95% Credible Interval | | | |
|  | | Group | | N | | Mean | | SD | | SE | | Coefficient of variation | | Lower | | Upper | |
| cortisol.nmol.L |  | TM |  | 11 |  | 2.491 |  | 1.104 |  | 0.333 |  | 0.443 |  | 1.749 |  | 3.233 |  |
|  |  | TF |  | 7 |  | 1.743 |  | 0.735 |  | 0.278 |  | 0.421 |  | 1.064 |  | 2.422 |  |
|  | | | | | | | | | | | | | | | | | |

### Sample 3

| *Bayesian Independent Samples T-Test* | | | | | |
| --- | --- | --- | --- | --- | --- |
|  | | BF₊₀ | | error % | |
| cortisol.nmol.L |  | 0.253 |  | ~ 2.175×10^-4^ |  |
|  | | | | | |
| *Note.*  For all tests, the alternative hypothesis specifies that the location of group *TM* is greater than the location of group *TF* . | | | | | |

**cortisol.nmol.L**

**Prior and Posterior**


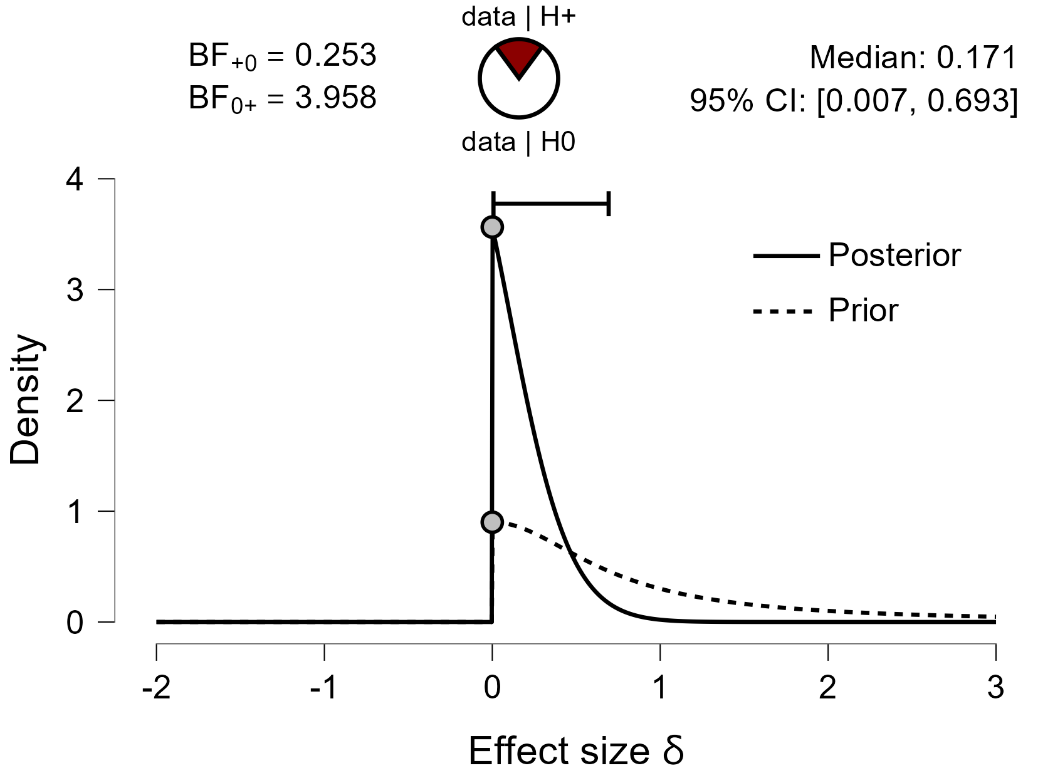


**Bayes Factor Robustness Check**


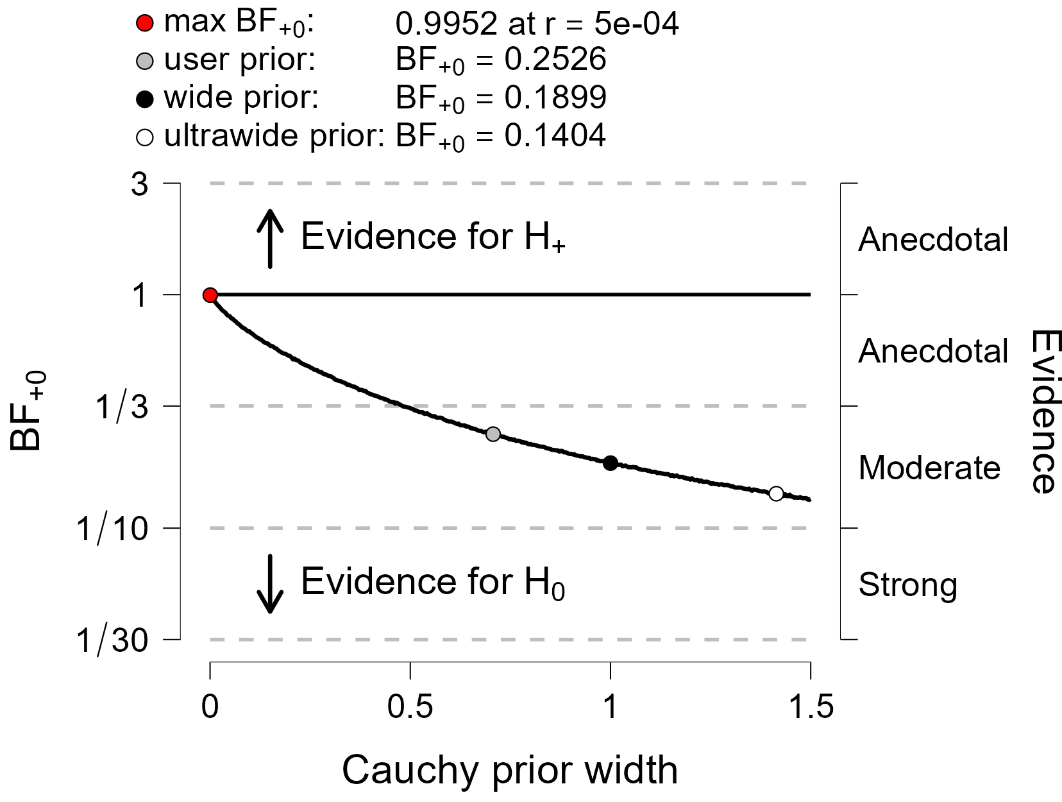


| *Descriptives* | | | | | | | | | | | | | | | | | |
| --- | --- | --- | --- | --- | --- | --- | --- | --- | --- | --- | --- | --- | --- | --- | --- | --- | --- |
|  | | | | | | | | | | | | | | 95% Credible Interval | | | |
|  | | Group | | N | | Mean | | SD | | SE | | Coefficient of variation | | Lower | | Upper | |
| cortisol.nmol.L |  | TM |  | 11 |  | 1.541 |  | 1.239 |  | 0.374 |  | 0.804 |  | 0.708 |  | 2.373 |  |
|  |  | TF |  | 7 |  | 2.371 |  | 2.424 |  | 0.916 |  | 1.022 |  | 0.130 |  | 4.613 |  |
|  | | | | | | | | | | | | | | | | | |

### Sample 4

| *Bayesian Independent Samples T-Test* | | | | | |
| --- | --- | --- | --- | --- | --- |
|  | | BF₊₀ | | error % | |
| cortisol.nmol.L |  | 1.055 |  | ~ 0.016 |  |
|  | | | | | |
| *Note.*  For all tests, the alternative hypothesis specifies that the location of group *TM* is greater than the location of group *TF* . | | | | | |

**cortisol.nmol.L**

- **Prior and Posterior**
-
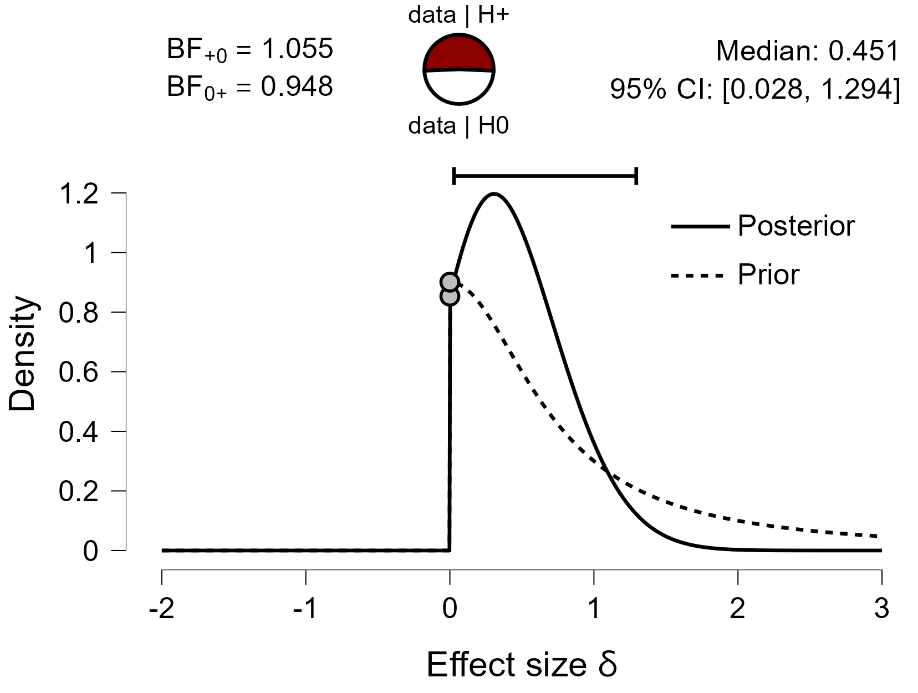

- **Bayes Factor Robustness Check**
-
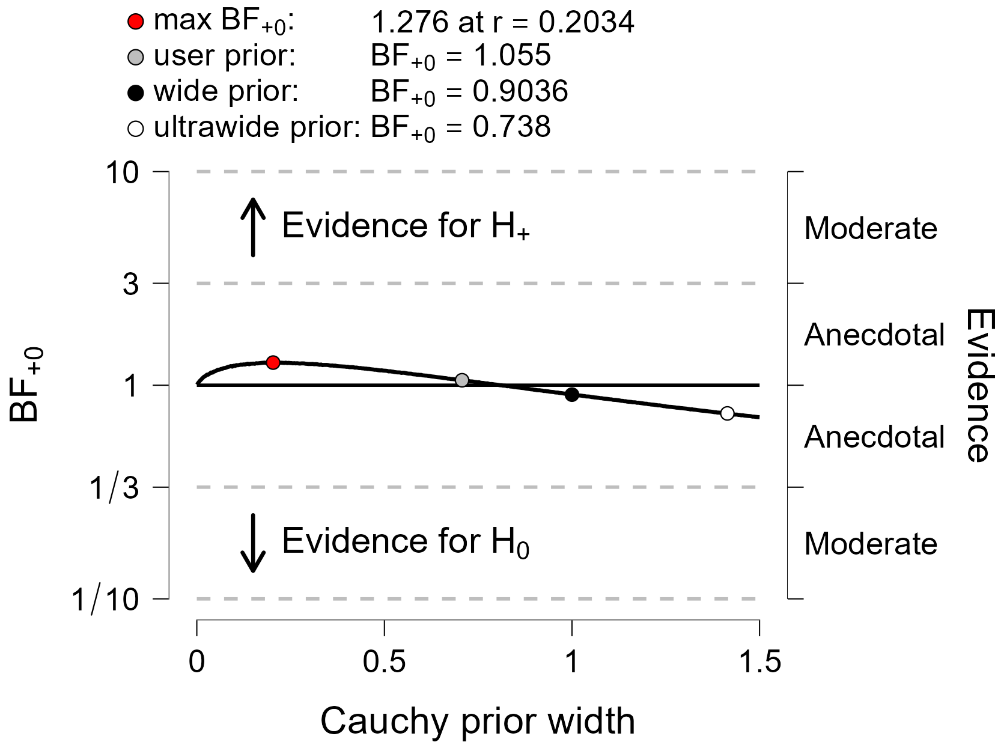


| *Descriptives* | | | | | | | | | | | | | | | | | |
| --- | --- | --- | --- | --- | --- | --- | --- | --- | --- | --- | --- | --- | --- | --- | --- | --- | --- |
|  | | | | | | | | | | | | | | 95% Credible Interval | | | |
|  | | Group | | N | | Mean | | SD | | SE | | Coefficient of variation | | Lower | | Upper | |
| cortisol.nmol.L |  | TM |  | 11 |  | 0.650 |  | 0.475 |  | 0.143 |  | 0.731 |  | 0.331 |  | 0.969 |  |
|  |  | TF |  | 7 |  | 0.429 |  | 0.240 |  | 0.091 |  | 0.559 |  | 0.207 |  | 0.650 |  |
|  | | | | | | | | | | | | | | | | | |

### Total cortisol mean

| *Bayesian Independent Samples T-Test* | | | | | |
| --- | --- | --- | --- | --- | --- |
|  | | BF₊₀ | | error % | |
| MeanCortAllDay |  | 1.497 |  | ~ 7.662×10^-6^ |  |
|  | | | | | |
| Note.  For all tests, the alternative hypothesis specifies that the location of group TM is greater than the location of group TF . | | | | | |

 MeanCortAllDay


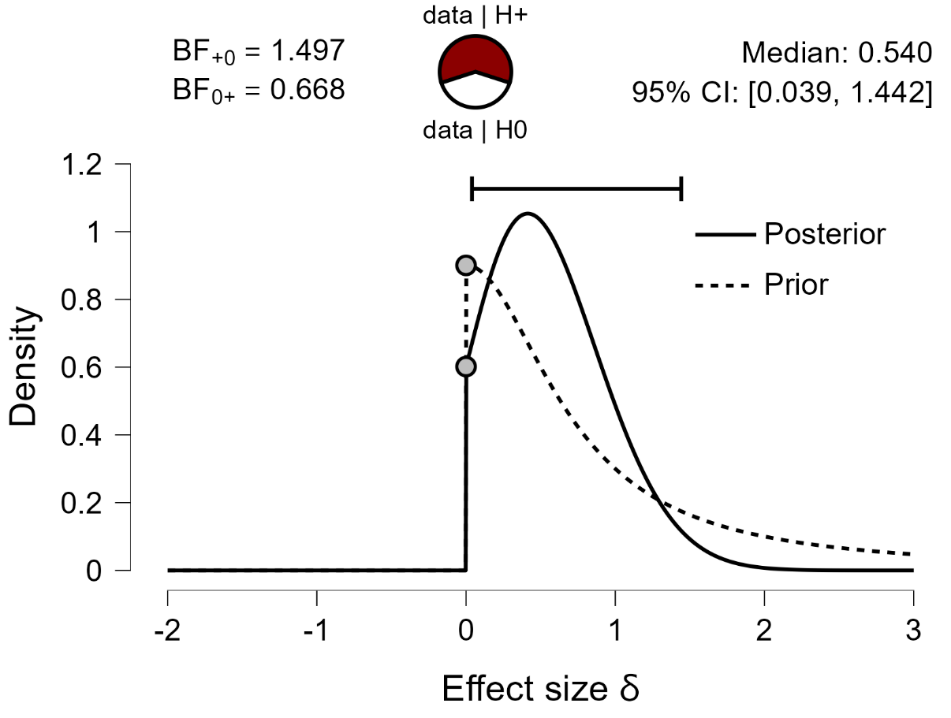


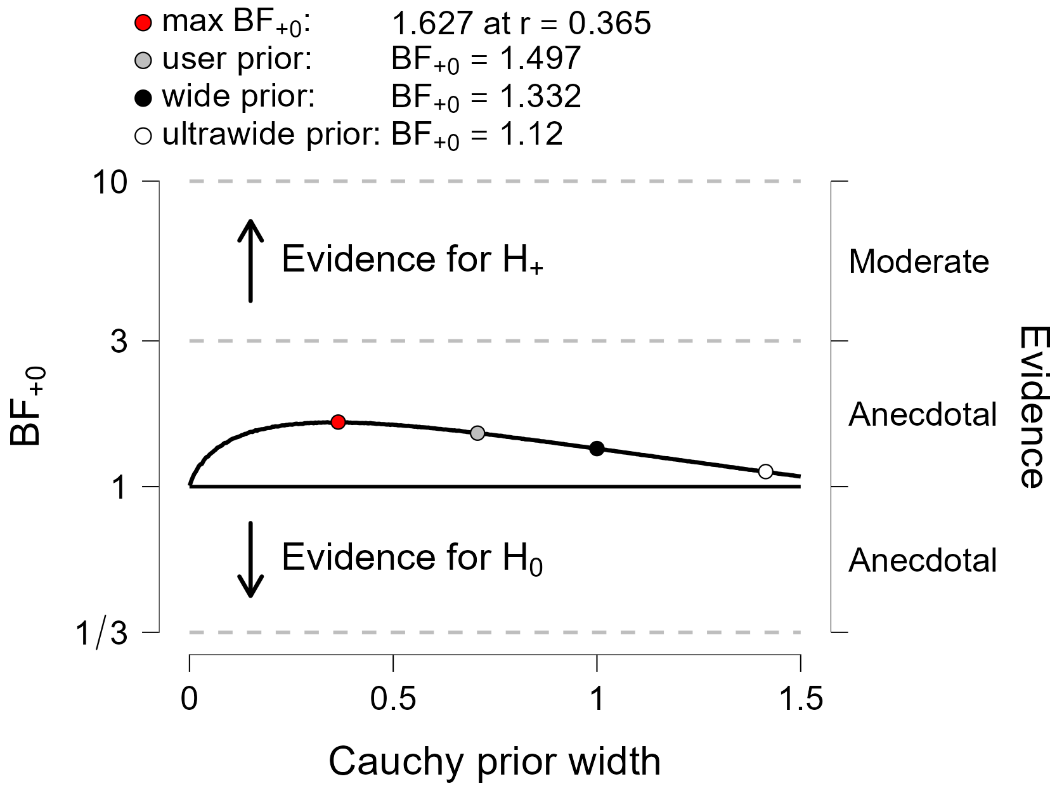


| *Descriptives* | | | | | | | | | | | | | | | | | |
| --- | --- | --- | --- | --- | --- | --- | --- | --- | --- | --- | --- | --- | --- | --- | --- | --- | --- |
|  | | | | | | | | | | | | | | 95% Credible Interval | | | |
|  | | Group | | N | | Mean | | SD | | SE | | Coefficient of variation | | Lower | | Upper | |
| cortisol.nmol.L |  | TM |  | 11 |  | 11.195 |  | 6.924 |  | 2.088 |  | 0.618 |  | 6.544 |  | 15.847 |  |
|  |  | TF |  | 7 |  | 6.343 |  | 3.198 |  | 1.209 |  | 0.504 |  | 3.385 |  | 9.300 |  |

### AUCg

| *Bayesian Independent Samples T-Test* | | | | | |
| --- | --- | --- | --- | --- | --- |
|  | | BF₊₀ | | error % | |
| AUCg |  | 1.257 |  | ~ 0.009 |  |
|  | | | | | |
| Note.  For all tests, the alternative hypothesis specifies that the location of group TM is greater than the location of group TF . | | | | | |

##### Prior and Posterior


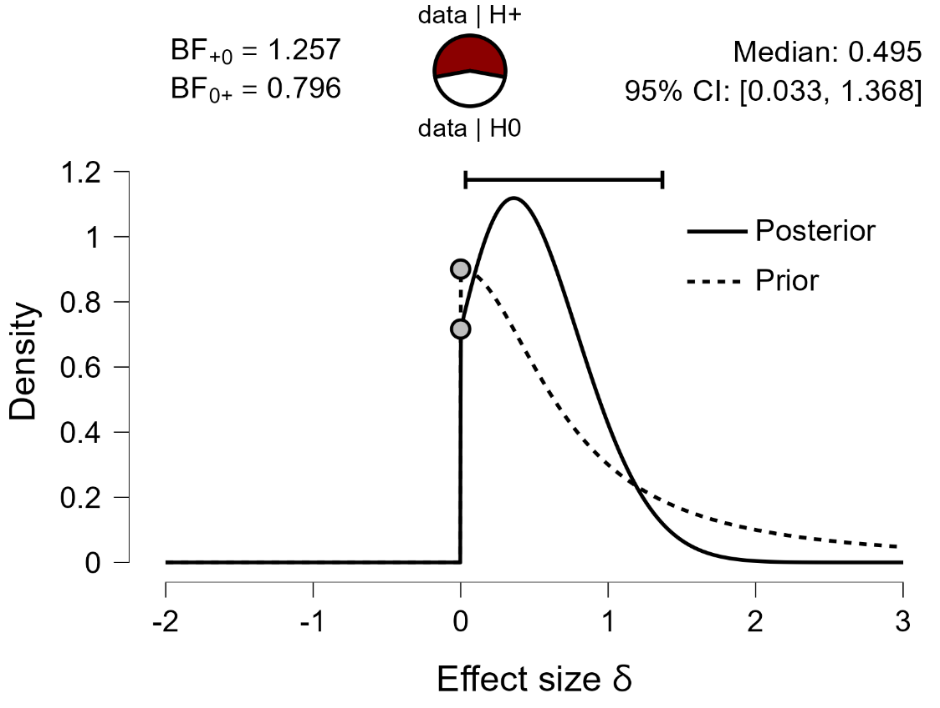


##### Bayes Factor Robustness Check


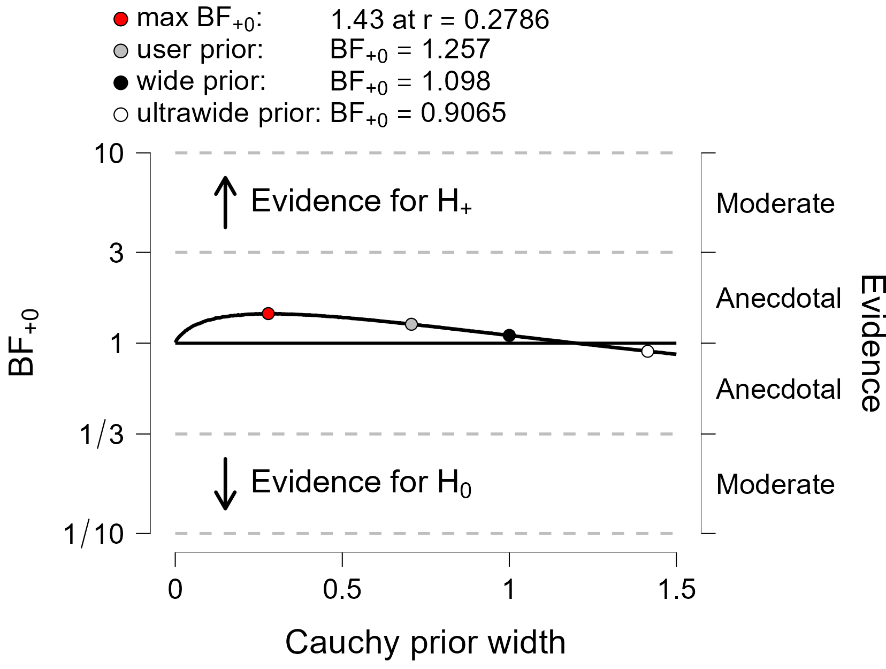


| *Descriptives* | | | | | | | | | | | | | | | | | |
| --- | --- | --- | --- | --- | --- | --- | --- | --- | --- | --- | --- | --- | --- | --- | --- | --- | --- |
|  | | | | | | | | | | | | | | 95% Credible Interval | | | |
|  | | Group | | N | | Mean | | SD | | SE | | Coefficient of variation | | Lower | | Upper | |
| AUCg |  | TM |  | 11 |  | 50.494 |  | 25.147 |  | 7.582 |  | 0.498 |  | 33.600 |  | 67.388 |  |
|  |  | TF |  | 7 |  | 36.760 |  | 14.315 |  | 5.411 |  | 0.389 |  | 23.521 |  | 50.000 |  |
|  | | | | | | | | | | | | | | | | | |

### T4 to T1 slope

| *Bayesian Independent Samples T-Test* | | | | | |
| --- | --- | --- | --- | --- | --- |
|  | | BF₋₀ | | error % | |
| CortT4toT1 |  | 1.989 |  | ~ 9.384×10^-6^ |  |
|  | | | | | |
| *Note.*  For all tests, the alternative hypothesis specifies that the location of group *TM* is smaller than the location of group *TF* . | | | | | |

**Prior and Posterior**


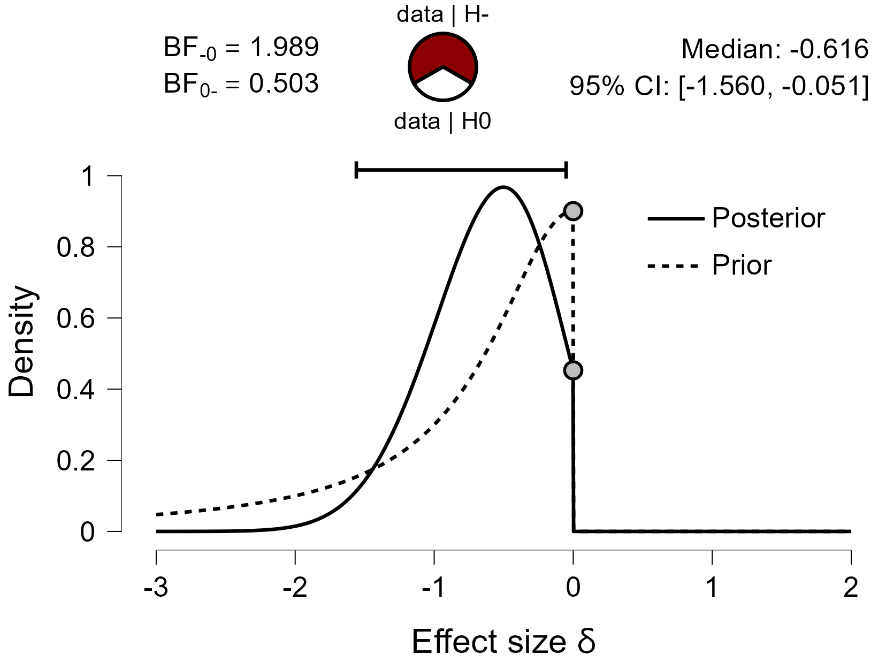


**Bayes Factor Robustness Check**


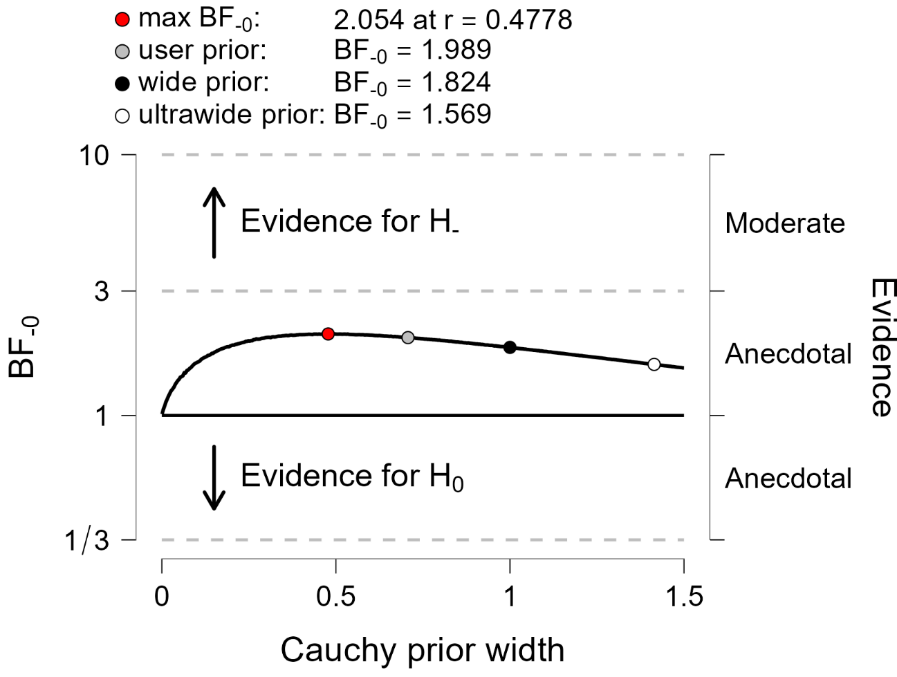


| *Descriptives* | | | | | | | | | | | | | | | | | |
| --- | --- | --- | --- | --- | --- | --- | --- | --- | --- | --- | --- | --- | --- | --- | --- | --- | --- |
|  | | | | | | | | | | | | | | 95% Credible Interval | | | |
|  | | Group | | N | | Mean | | SD | | SE | | Coefficient of variation | | Lower | | Upper | |
| CortT4toT1 |  | TM |  | 11 |  | -10.545 |  | 6.600 |  | 1.990 |  | -0.626 |  | -14.980 |  | -6.111 |  |
|  |  | TF |  | 7 |  | -5.914 |  | 3.208 |  | 1.213 |  | -0.542 |  | -8.881 |  | -2.947 |  |
|  | | | | | | | | | | | | | | | | | |

## Hypothesis 2: TM group

We hypothesize that the transmasculine group shows decreases in absolute salivary cortisol levels, mean cortisol level, AUCg and an increase in the T4 to T1 slope after 3 months of GAHT.

### Sample 1

| *Bayesian Paired Samples T-Test* | | | | | | | | | |
| --- | --- | --- | --- | --- | --- | --- | --- | --- | --- |
| Measure 1 | |  | | Measure 2 | | BF₊₀ | | error % | |
| Cortisol1.Base |  | - |  | Cortisol1.3MO |  | 0.253 |  | ~ 3.521×10^-5^ |  |
|  | | | | | | | | | |
| *Note.*  For all tests, the alternative hypothesis specifies that Measure 1 is greater than Measure 2. For example, Cortisol1.Base is greater than Cortisol1.3MO. | | | | | | | | | |

**Cortisol1.Base - Cortisol1.3MO**

**Prior and Posterior**


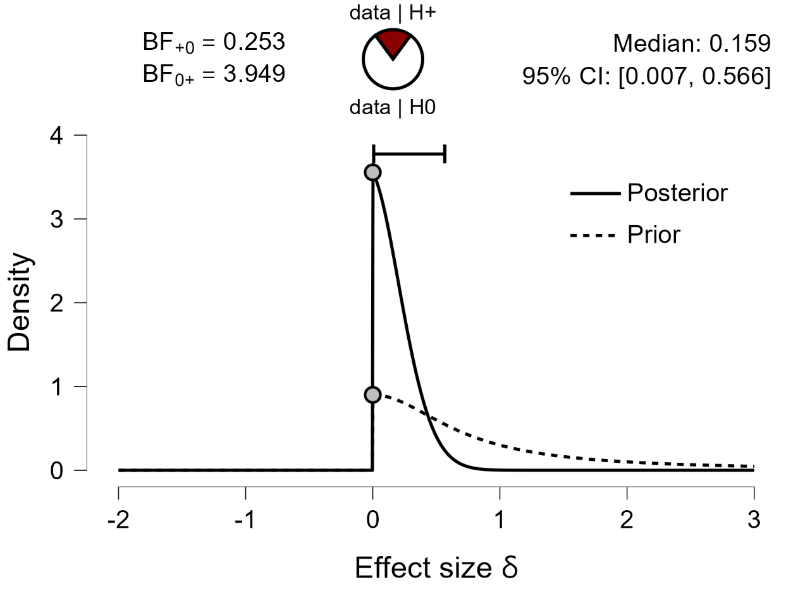


**Bayes Factor Robustness Check**


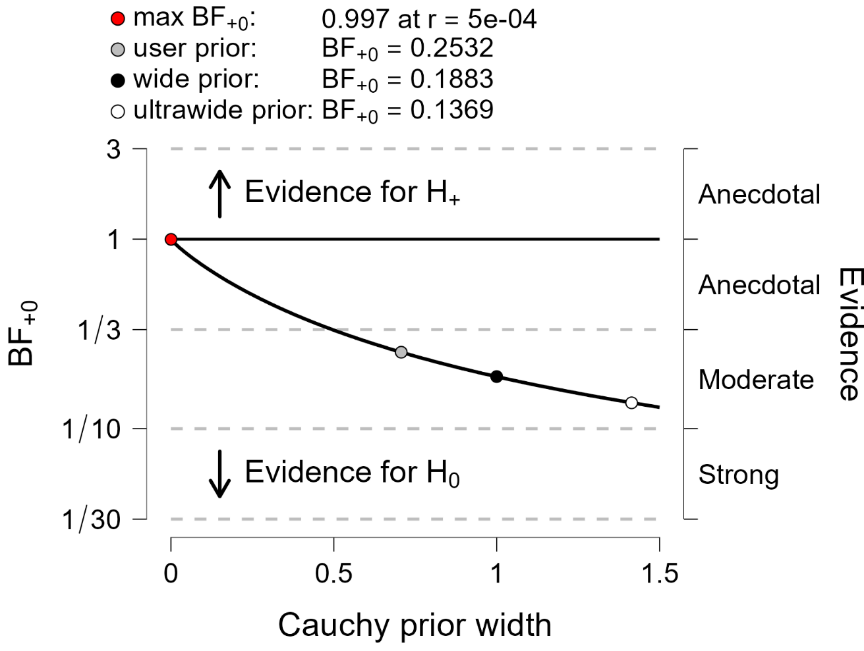


| *Descriptives* | | | | | | | | | | | | | | | |
| --- | --- | --- | --- | --- | --- | --- | --- | --- | --- | --- | --- | --- | --- | --- | --- |
|  | | | | | | | | | | | | 95% Credible Interval | | | |
|  | | N | | Mean | | SD | | SE | | Coefficient of variation | | Lower | | Upper | |
| Cortisol1.Base |  | 11 |  | 11.195 |  | 6.924 |  | 2.088 |  | 0.618 |  | 6.544 |  | 15.847 |  |
| Cortisol1.3MO |  | 11 |  | 12.082 |  | 7.918 |  | 2.387 |  | 0.655 |  | 6.763 |  | 17.401 |  |
|  | | | | | | | | | | | | | | | |

### Sample 2

| *Bayesian Paired Samples T-Test* | | | | | | | | | |
| --- | --- | --- | --- | --- | --- | --- | --- | --- | --- |
| Measure 1 | |  | | Measure 2 | | BF₊₀ | | error % | |
| Cortisol2.Base |  | - |  | Cortisol2.3MO |  | 0.209 |  | ~ 8.508×10^-4^ |  |
|  | | | | | | | | | |
| *Note.*  For all tests, the alternative hypothesis specifies that Measure 1 is greater than Measure 2. For example, Cortisol1.Base is greater than Cortisol1.3MO. | | | | | | | | | |

**Cortisol2.Base - Cortisol2.3MO**

**Prior and Posterior**


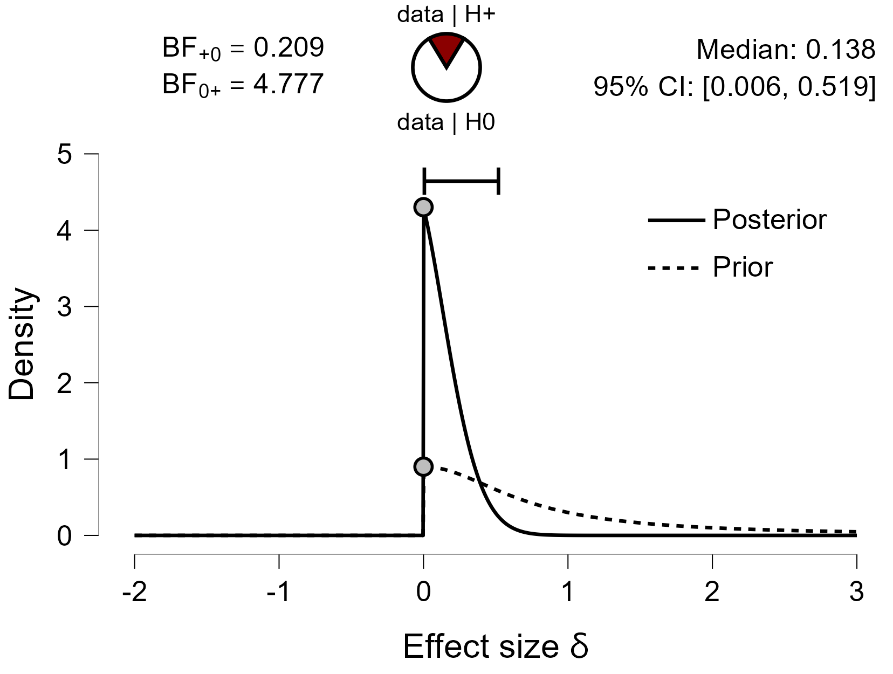


**Bayes Factor Robustness Check**


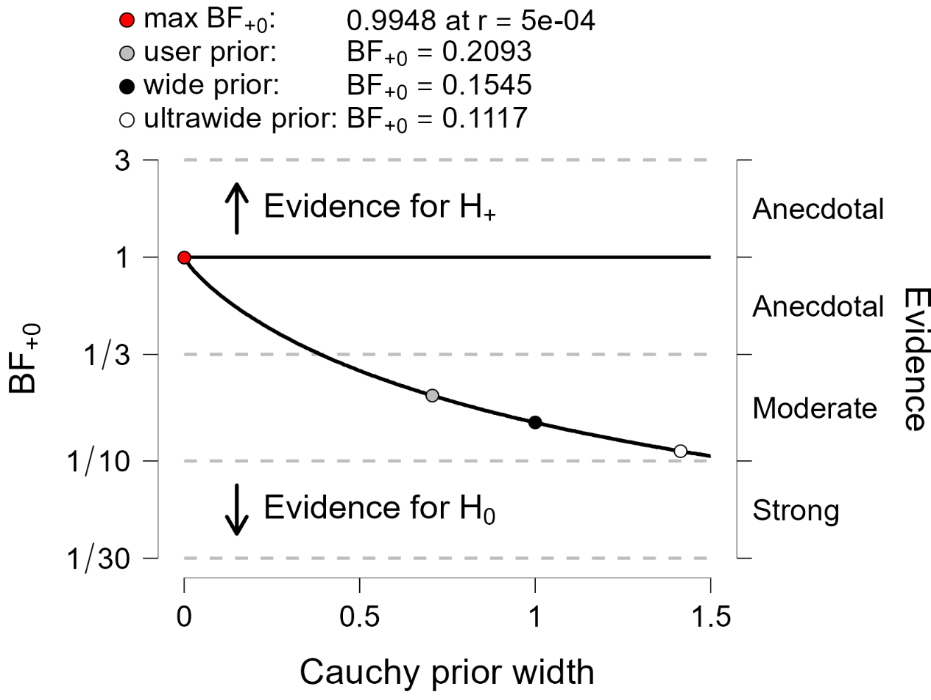


| *Descriptives* | | | | | | | | | | | | | | | |
| --- | --- | --- | --- | --- | --- | --- | --- | --- | --- | --- | --- | --- | --- | --- | --- |
|  | | | | | | | | | | | | 95% Credible Interval | | | |
|  | | N | | Mean | | SD | | SE | | Coefficient of variation | | Lower | | Upper | |
| Cortisol2.Base |  | 11 |  | 2.491 |  | 1.104 |  | 0.333 |  | 0.443 |  | 1.749 |  | 3.233 |  |
| Cortisol2.3MO |  | 11 |  | 2.991 |  | 2.388 |  | 0.720 |  | 0.798 |  | 1.387 |  | 4.595 |  |
|  | | | | | | | | | | | | | | | |

### Sample 3

| *Bayesian Paired Samples T-Test* | | | | | | | | | |
| --- | --- | --- | --- | --- | --- | --- | --- | --- | --- |
| Measure 1 | |  | | Measure 2 | | BF₊₀ | | error % | |
| Cortisol3.Base |  | - |  | Cortisol3.3MO |  | 0.442 |  | ~ 3.346×10^-6^ |  |
|  | | | | | | | | | |
| *Note.*  For all tests, the alternative hypothesis specifies that Measure 1 is greater than Measure 2. For example, Cortisol1.Base is greater than Cortisol1.3MO. | | | | | | | | | |

**Cortisol3.Base - Cortisol3.3MO**

**Prior and Posterior**


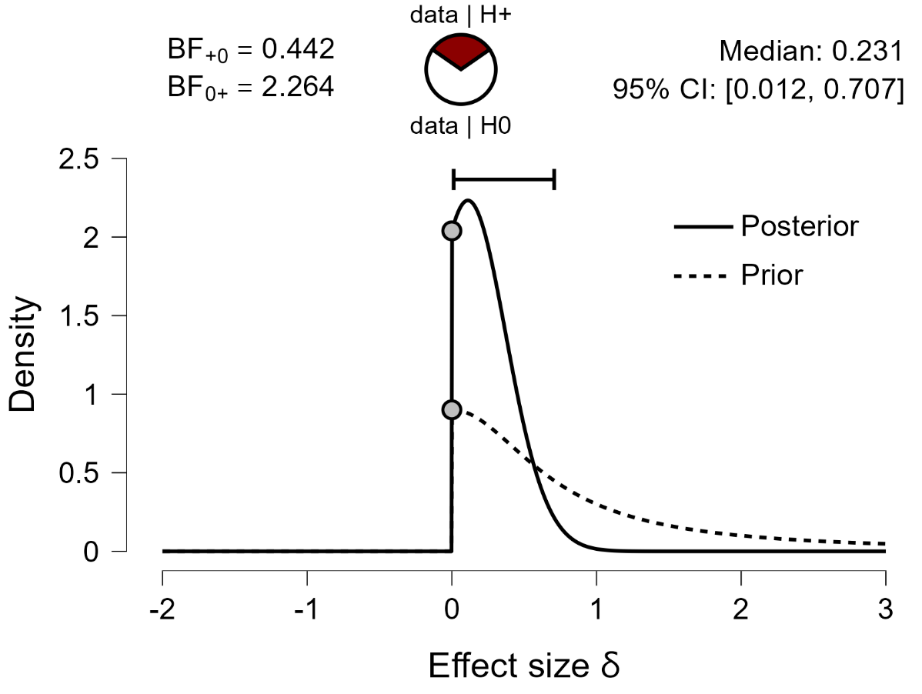


**Bayes Factor Robustness Check**


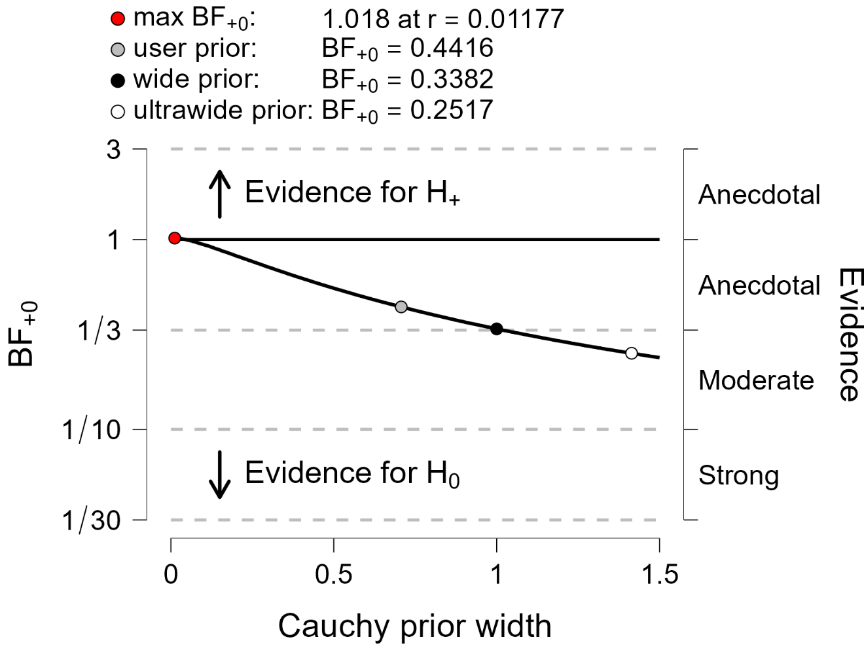


| *Descriptives* | | | | | | | | | | | | | | | |
| --- | --- | --- | --- | --- | --- | --- | --- | --- | --- | --- | --- | --- | --- | --- | --- |
|  | | | | | | | | | | | | 95% Credible Interval | | | |
|  | | N | | Mean | | SD | | SE | | Coefficient of variation | | Lower | | Upper | |
| Cortisol3.Base |  | 11 |  | 1.541 |  | 1.239 |  | 0.374 |  | 0.804 |  | 0.708 |  | 2.373 |  |
| Cortisol3.3MO |  | 11 |  | 1.255 |  | 1.174 |  | 0.354 |  | 0.936 |  | 0.466 |  | 2.043 |  |
|  | | | | | | | | | | | | | | | |

### Sample 4

| *Bayesian Paired Samples T-Test* | | | | | | | | | |
| --- | --- | --- | --- | --- | --- | --- | --- | --- | --- |
| Measure 1 | |  | | Measure 2 | | BF₊₀ | | error % | |
| Cortisol4.Base |  | - |  | Cortisol4.3MO |  | 0.177 |  | ~ 6.486×10^-4^ |  |
|  | | | | | | | | | |
| *Note.*  For all tests, the alternative hypothesis specifies that Measure 1 is greater than Measure 2. For example, Cortisol1.Base is greater than Cortisol1.3MO. | | | | | | | | | |

**Cortisol4.Base - Cortisol4.3MO**

**Prior and Posterior**


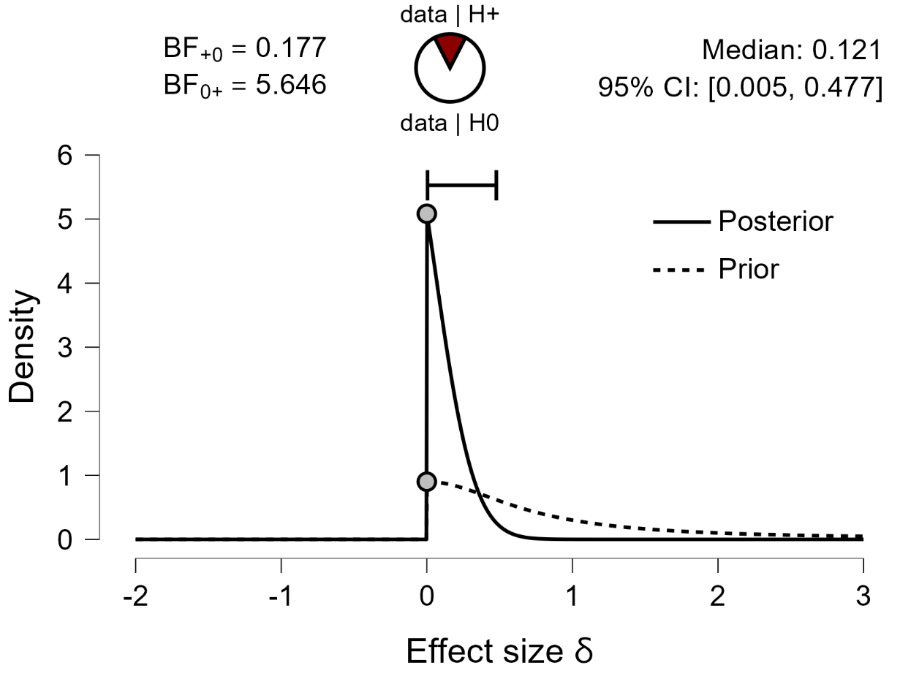


**Bayes Factor Robustness Check**


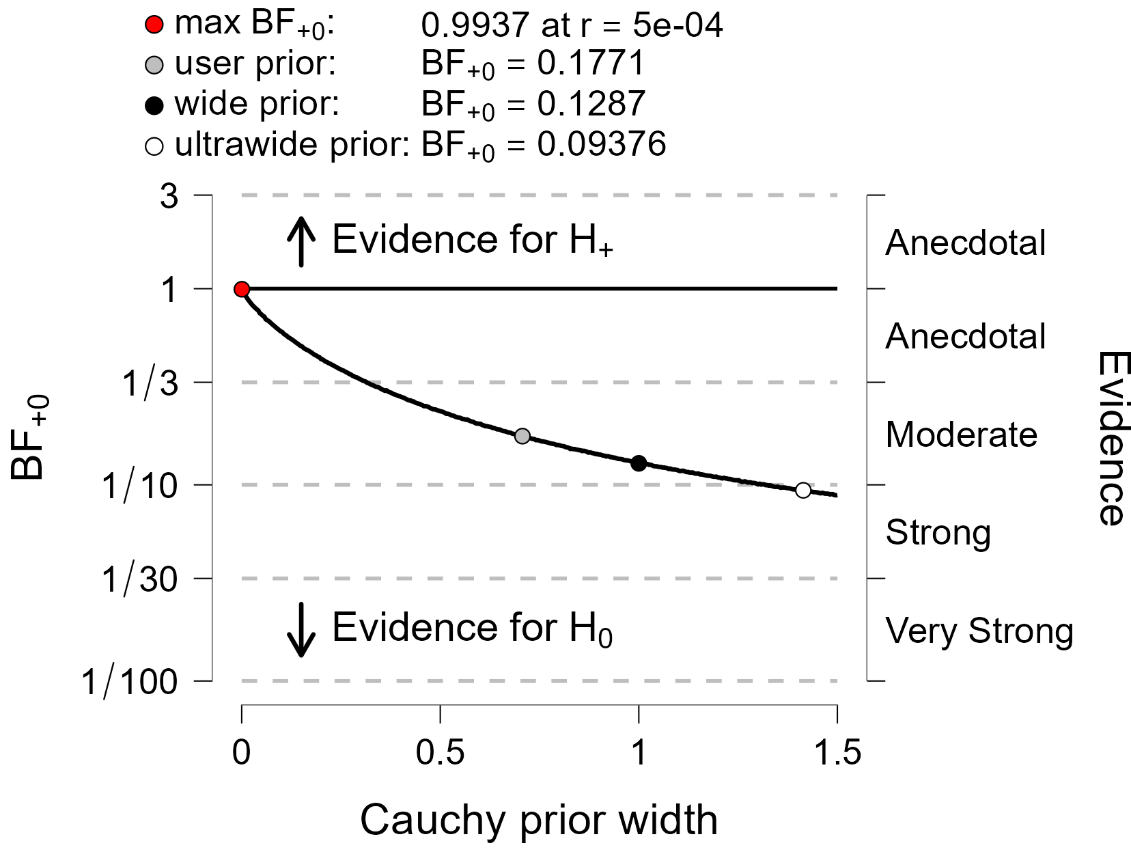


| *Descriptives* | | | | | | | | | | | | | | | |
| --- | --- | --- | --- | --- | --- | --- | --- | --- | --- | --- | --- | --- | --- | --- | --- |
|  | | | | | | | | | | | | 95% Credible Interval | | | |
|  | | N | | Mean | | SD | | SE | | Coefficient of variation | | Lower | | Upper | |
| Cortisol4.Base |  | 11 |  | 0.650 |  | 0.475 |  | 0.143 |  | 0.731 |  | 0.331 |  | 0.969 |  |
| Cortisol4.3MO |  | 11 |  | 1.082 |  | 1.458 |  | 0.440 |  | 1.348 |  | 0.102 |  | 2.062 |  |
|  | | | | | | | | | | | | | | | |

### Total cortisol mean

| *Bayesian Paired Samples T-Test* | | | | | | | | | |
| --- | --- | --- | --- | --- | --- | --- | --- | --- | --- |
| Measure 1 | |  | | Measure 2 | | BF₊₀ | | error % | |
| MeanCortAllDay.Base |  | - |  | MeanCortAllDay.3MO |  | 0.243 |  | ~ 2.578×10^-4^ |  |
|  | | | | | | | | | |
| *Note.*  For all tests, the alternative hypothesis specifies that Measure 1 is greater than Measure 2. For example, MeanCortAllDay.Base is greater than MeanCortAllDay.3MO. | | | | | | | | | |

**MeanCortAllDay.Base - MeanCortAllDay.3MO**

**Prior and Posterior**


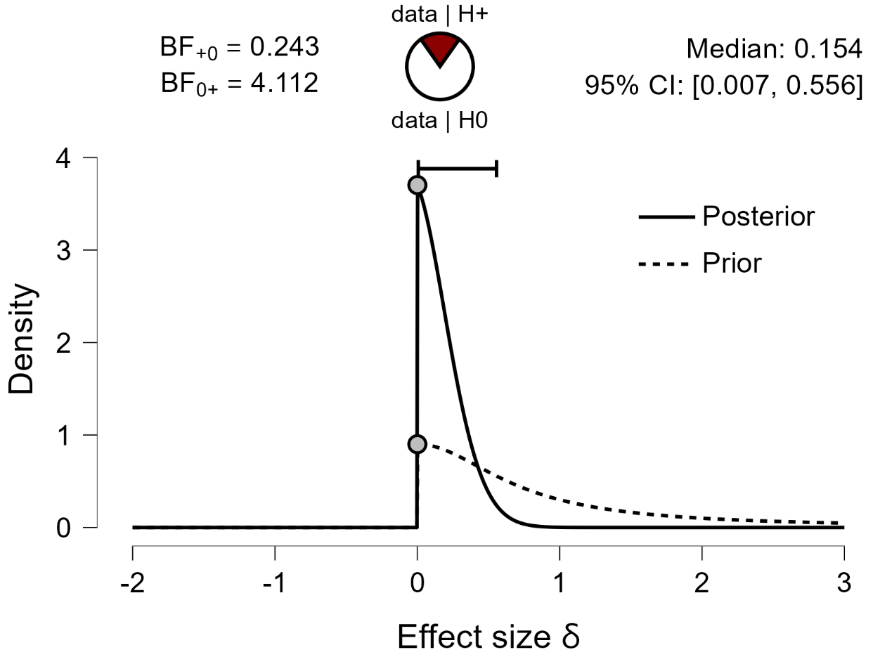


**Bayes Factor Robustness Check**


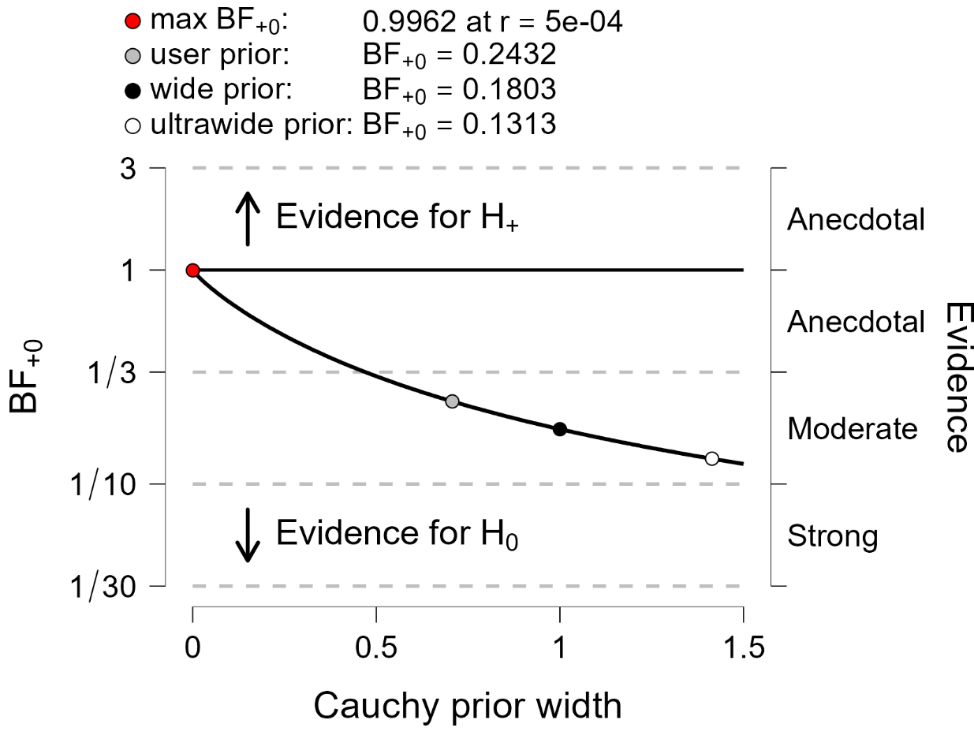


| *Descriptives* | | | | | | | | | | | | | | | |
| --- | --- | --- | --- | --- | --- | --- | --- | --- | --- | --- | --- | --- | --- | --- | --- |
|  | | | | | | | | | | | | 95% Credible Interval | | | |
|  | | N | | Mean | | SD | | SE | | Coefficient of variation | | Lower | | Upper | |
| MeanCortAllDay.Base |  | 11 |  | 3.969 |  | 2.109 |  | 0.636 |  | 0.531 |  | 2.552 |  | 5.386 |  |
| MeanCortAllDay.3MO |  | 11 |  | 4.352 |  | 2.714 |  | 0.818 |  | 0.624 |  | 2.529 |  | 6.175 |  |
|  | | | | | | | | | | | | | | | |

### AUCg

| *Bayesian Paired Samples T-Test* | | | | | | | | | |
| --- | --- | --- | --- | --- | --- | --- | --- | --- | --- |
| Measure 1 | |  | | Measure 2 | | BF₊₀ | | error % | |
| AUCg.Base |  | - |  | AUCg.3MO |  | 0.260 |  | ~ 4.393×10^-5^ |  |
|  | | | | | | | | | |
| *Note.*  For all tests, the alternative hypothesis specifies that Measure 1 is greater than Measure 2. For example, MeanCortAllDay.Base is greater than MeanCortAllDay.3MO. | | | | | | | | | |

**AUCg.Base - AUCg.3MO**

**Prior and Posterior**


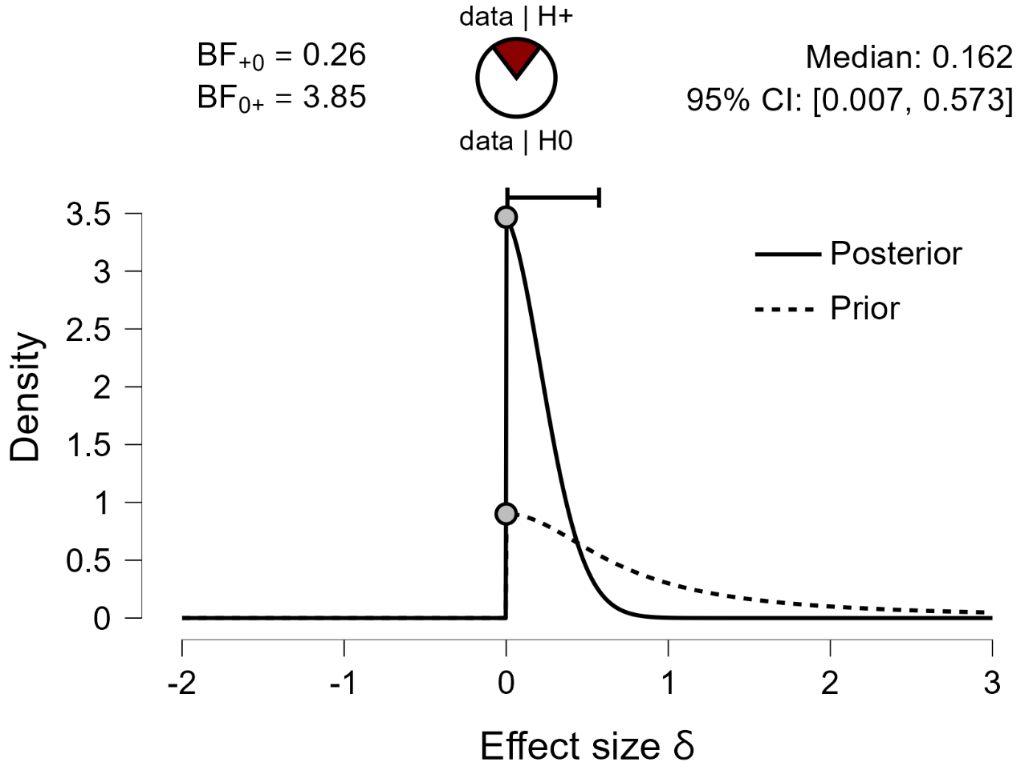


**Bayes Factor Robustness Check**


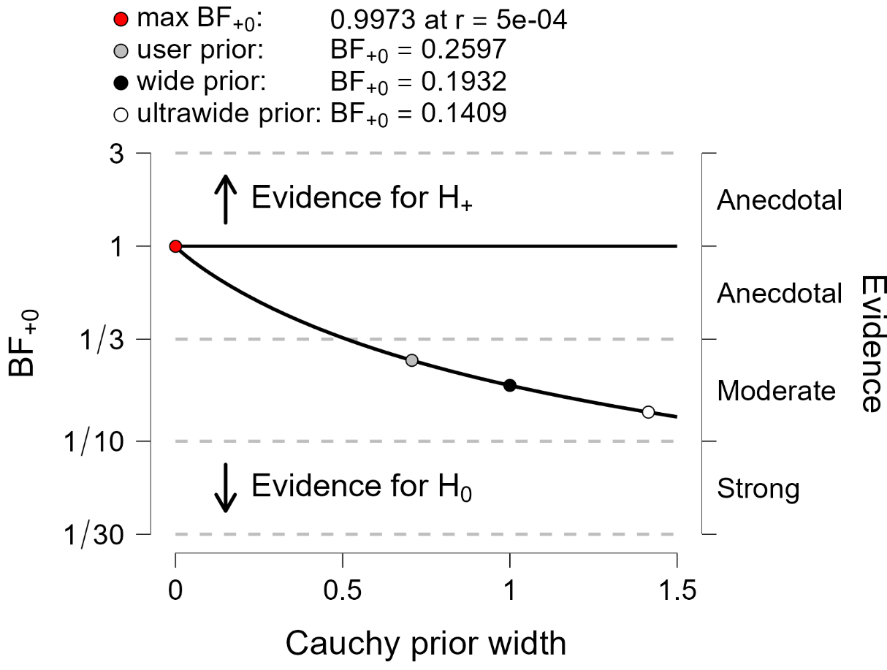


| *Descriptives* | | | | | | | | | | | | | | | |
| --- | --- | --- | --- | --- | --- | --- | --- | --- | --- | --- | --- | --- | --- | --- | --- |
|  | | | | | | | | | | | | 95% Credible Interval | | | |
|  | | N | | Mean | | SD | | SE | | Coefficient of variation | | Lower | | Upper | |
| AUCg.Base |  | 11 |  | 50.494 |  | 25.147 |  | 7.582 |  | 0.498 |  | 33.600 |  | 67.388 |  |
| AUCg.3MO |  | 11 |  | 53.463 |  | 31.357 |  | 9.454 |  | 0.587 |  | 32.397 |  | 74.529 |  |
|  | | | | | | | | | | | | | | | |

### T4 to T1 slope

| *Bayesian Paired Samples T-Test* | | | | | | | | | |
| --- | --- | --- | --- | --- | --- | --- | --- | --- | --- |
| Measure 1 | |  | | Measure 2 | | BF₋₀ | | error % | |
| CortT4toT1.Base |  | - |  | CortT4toT1.3MO |  | 0.271 |  | ~ 4.441×10^-4^ |  |
|  | | | | | | | | | |
| *Note.*  For all tests, the alternative hypothesis specifies that CortT4toT1.Base is less than CortT4toT1.3MO. | | | | | | | | | |

**CortT4toT1.Base - CortT4toT1.3MO**

**Prior and Posterior**


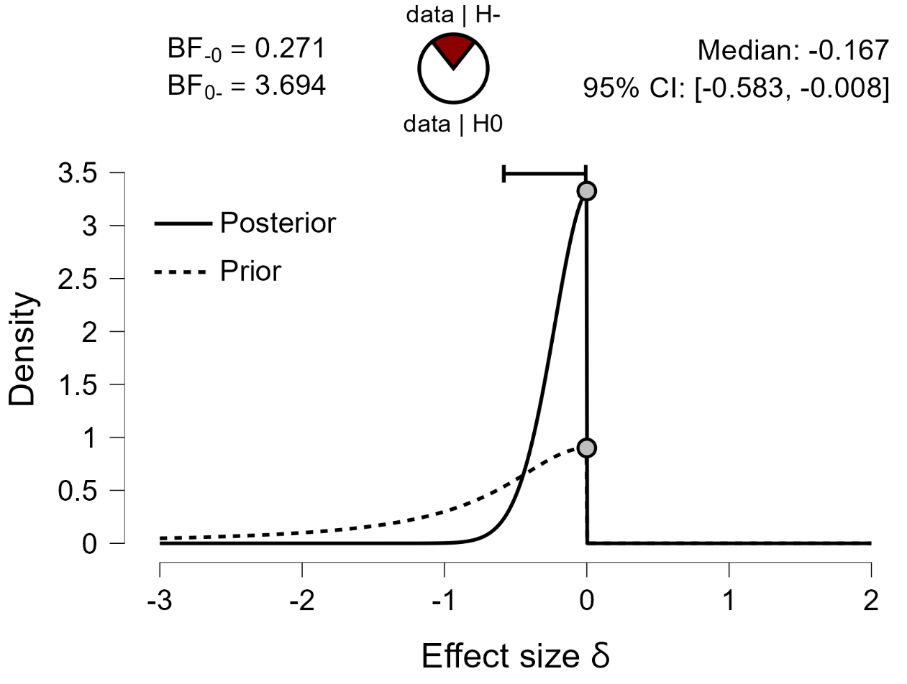


**Bayes Factor Robustness Check**


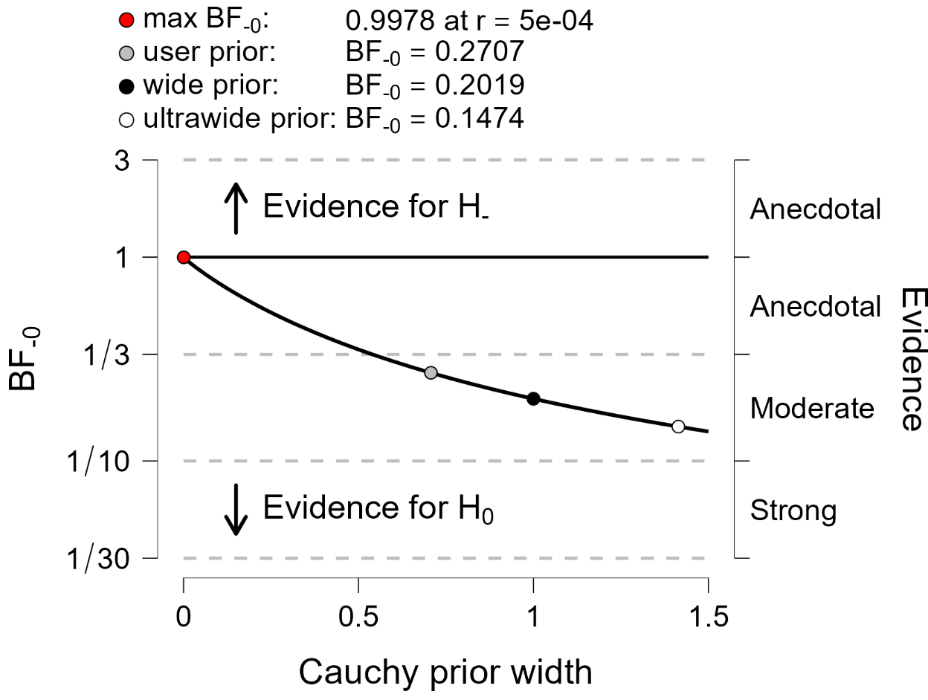


| *Descriptives* | | | | | | | | | | | | | | | |
| --- | --- | --- | --- | --- | --- | --- | --- | --- | --- | --- | --- | --- | --- | --- | --- |
|  | | | | | | | | | | | | 95% Credible Interval | | | |
|  | | N | | Mean | | SD | | SE | | Coefficient of variation | | Lower | | Upper | |
| CortT4toT1.Base |  | 11 |  | -10.545 |  | 6.600 |  | 1.990 |  | -0.626 |  | -14.980 |  | -6.111 |  |
| CortT4toT1.3MO |  | 11 |  | -11.000 |  | 6.838 |  | 2.062 |  | -0.622 |  | -15.594 |  | -6.406 |  |
|  | | | | | | | | | | | | | | | |

## Hypothesis 2: TF group

We hypothesize that the transfeminine group shows decreases in absolute salivary cortisol levels, mean cortisol level, AUCg and an increase in the T4 to T1 slope after 3 months of GAHT.

### Sample 1

| *Bayesian Paired Samples T-Test* | | | | | | | | | |
| --- | --- | --- | --- | --- | --- | --- | --- | --- | --- |
| Measure 1 | |  | | Measure 2 | | BF₋₀ | | error % | |
| Cortisol1.Base |  | - |  | Cortisol1.3MO |  | 1.112 |  | ~ 8.414×10^-6^ |  |
|  | | | | | | | | | |
| *Note.*  For all tests, the alternative hypothesis specifies that Measure 1 is less than Measure 2. For example, Cortisol1.Base is less than Cortisol1.3MO. | | | | | | | | | |

**Cortisol1.Base - Cortisol1.3MO**

**Prior and Posterior**


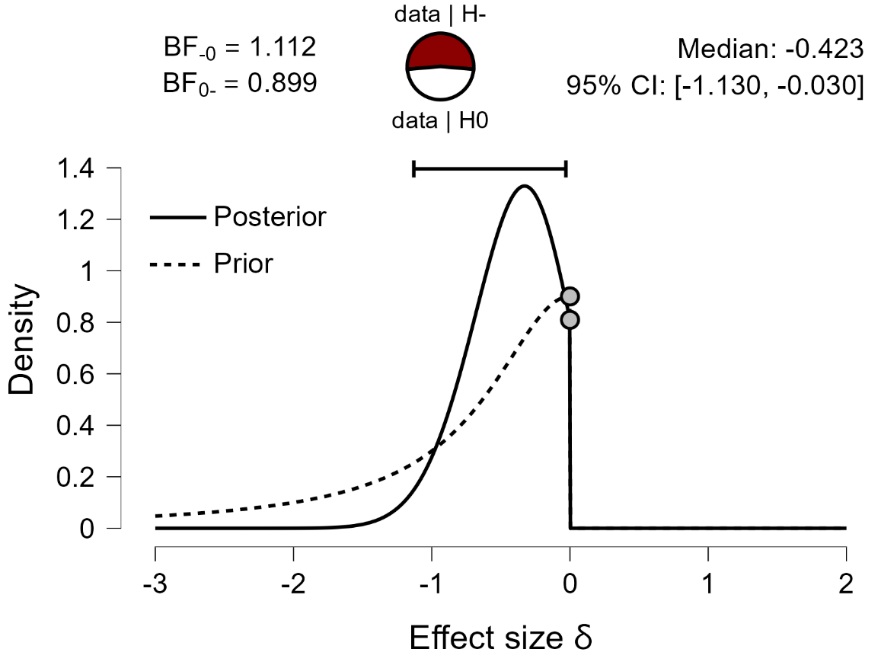


**Bayes Factor Robustness Check**


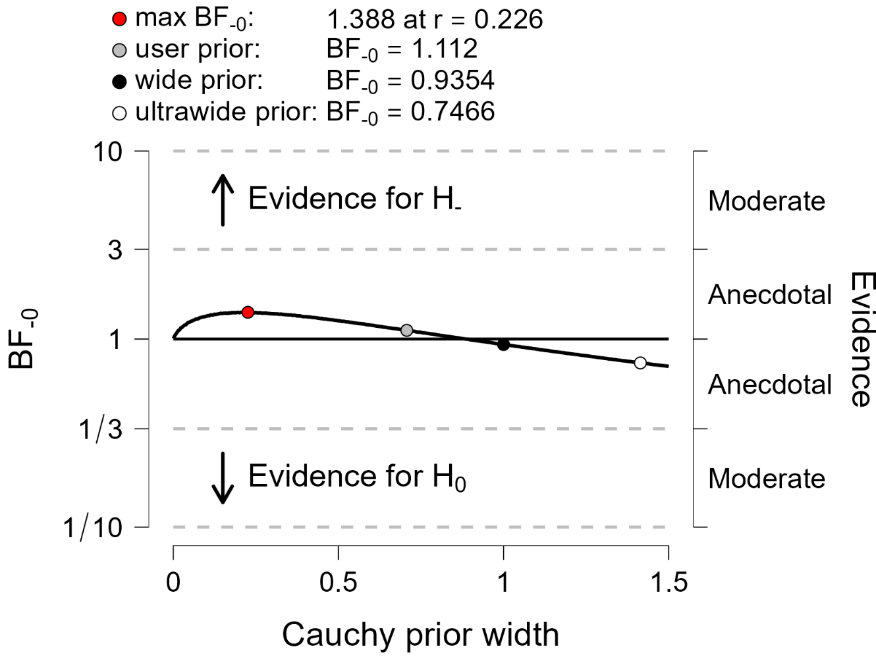


| *Descriptives* | | | | | | | | | | | | | | | |
| --- | --- | --- | --- | --- | --- | --- | --- | --- | --- | --- | --- | --- | --- | --- | --- |
|  | | | | | | | | | | | | 95% Credible Interval | | | |
|  | | N | | Mean | | SD | | SE | | Coefficient of variation | | Lower | | Upper | |
| Cortisol1.Base |  | 7 |  | 6.343 |  | 3.198 |  | 1.209 |  | 0.504 |  | 3.385 |  | 9.300 |  |
| Cortisol1.3MO |  | 7 |  | 8.929 |  | 5.149 |  | 1.946 |  | 0.577 |  | 4.167 |  | 13.691 |  |
|  | | | | | | | | | | | | | | | |

### Sample 2

| *Bayesian Paired Samples T-Test* | | | | | | | | | |
| --- | --- | --- | --- | --- | --- | --- | --- | --- | --- |
| Measure 1 | |  | | Measure 2 | | BF₋₀ | | error % | |
| Cortisol2.Base |  | - |  | Cortisol2.3MO |  | 3.236 |  | ~ 3.517×10^-4^ |  |
|  | | | | | | | | | |
| *Note.*  For all tests, the alternative hypothesis specifies that Measure 1 is less than Measure 2. For example, Cortisol1.Base is less than Cortisol1.3MO. | | | | | | | | | |

**Cortisol2.Base - Cortisol2.3MO**

**Prior and Posterior**


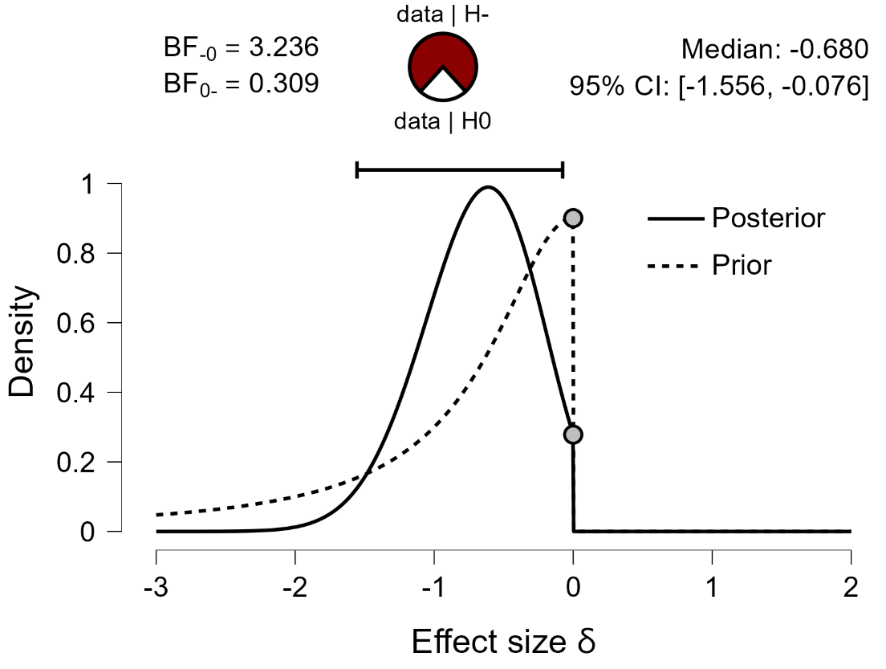


**Bayes Factor Robustness Check**


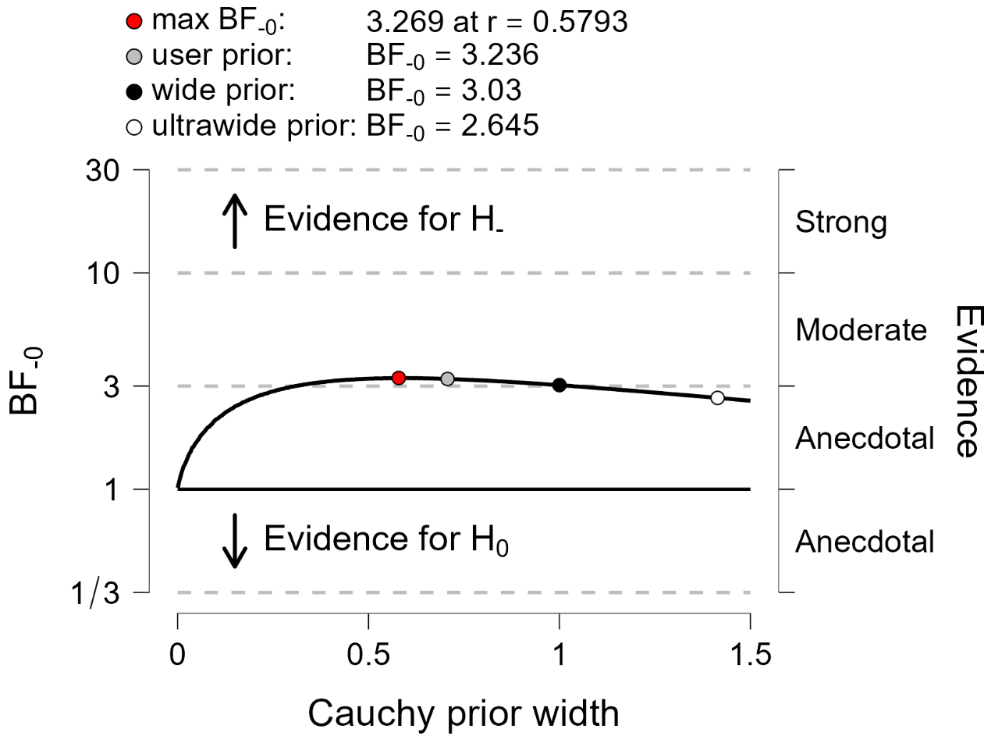


| *Descriptives* | | | | | | | | | | | | | | | |
| --- | --- | --- | --- | --- | --- | --- | --- | --- | --- | --- | --- | --- | --- | --- | --- |
|  | | | | | | | | | | | | 95% Credible Interval | | | |
|  | | N | | Mean | | SD | | SE | | Coefficient of variation | | Lower | | Upper | |
| Cortisol2.Base |  | 7 |  | 1.743 |  | 0.735 |  | 0.278 |  | 0.421 |  | 1.064 |  | 2.422 |  |
| Cortisol2.3MO |  | 7 |  | 2.471 |  | 1.135 |  | 0.429 |  | 0.459 |  | 1.421 |  | 3.521 |  |
|  | | | | | | | | | | | | | | | |

### Sample 3

| *Bayesian Paired Samples T-Test* | | | | | | | | | |
| --- | --- | --- | --- | --- | --- | --- | --- | --- | --- |
| Measure 1 | |  | | Measure 2 | | BF₋₀ | | error % | |
| Cortisol3.Base |  | - |  | Cortisol3.3MO |  | 0.616 |  | ~ 1.008×10^-6^ |  |
|  | | | | | | | | | |
| *Note.*  For all tests, the alternative hypothesis specifies that Measure 1 is less than Measure 2. For example, Cortisol1.Base is less than Cortisol1.3MO. | | | | | | | | | |

**Cortisol3.Base - Cortisol3.3MO**

**Prior and Posterior**


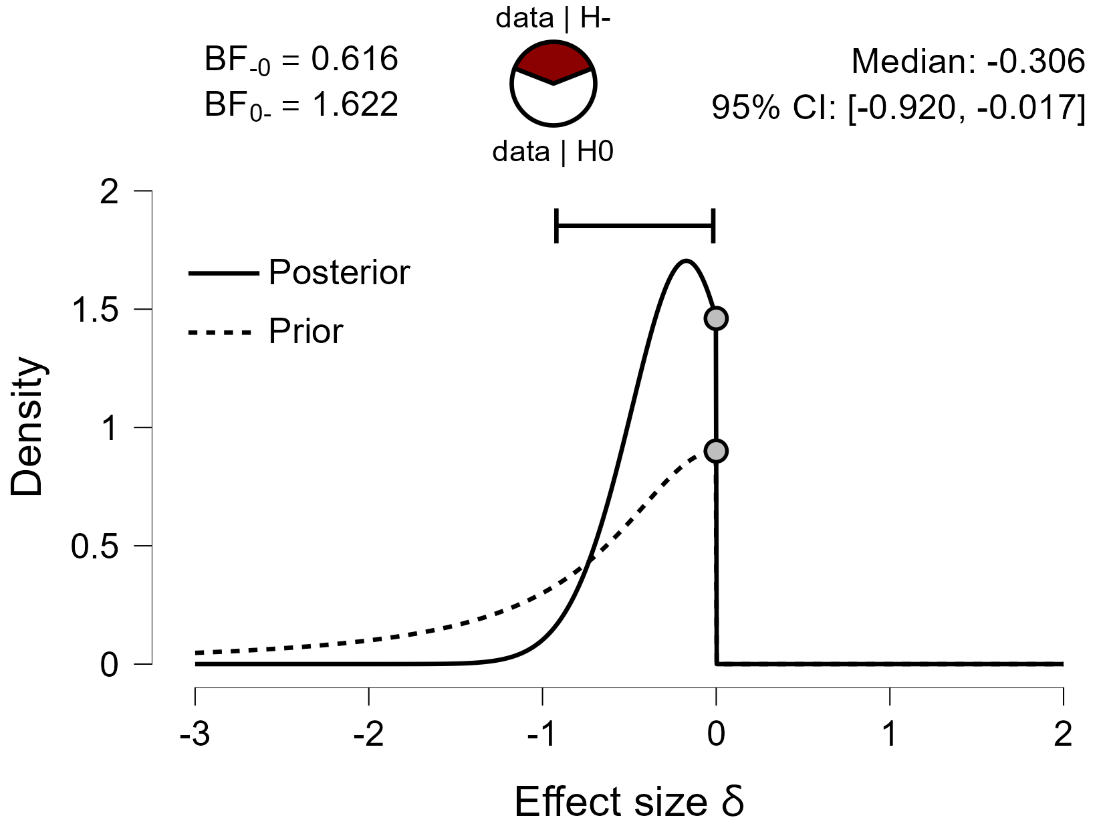


**Bayes Factor Robustness Check**


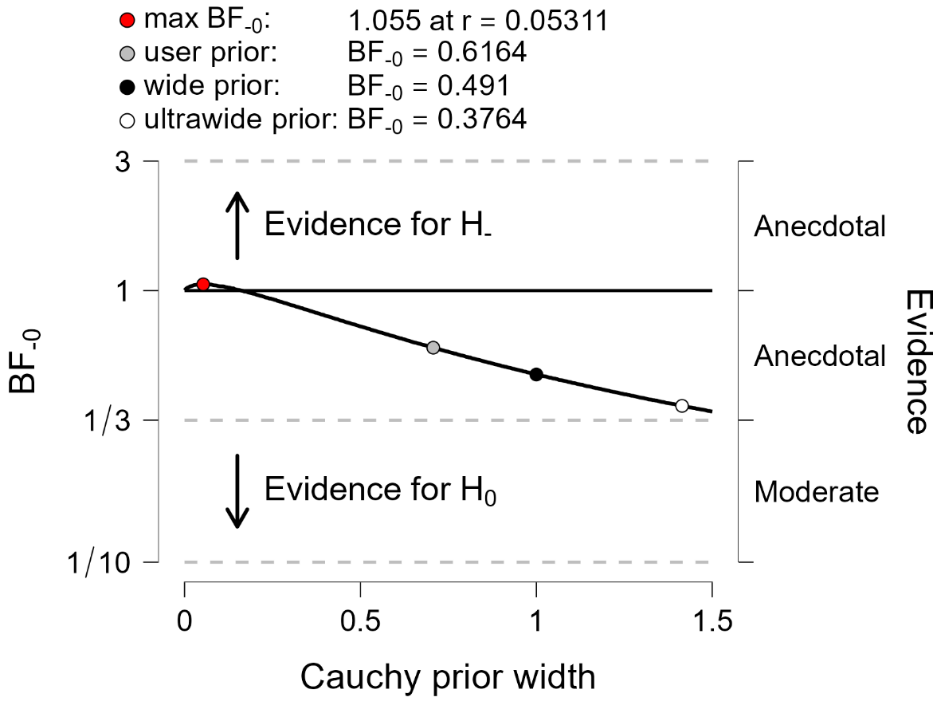


| *Descriptives* | | | | | | | | | | | | | | | |
| --- | --- | --- | --- | --- | --- | --- | --- | --- | --- | --- | --- | --- | --- | --- | --- |
|  | | | | | | | | | | | | 95% Credible Interval | | | |
|  | | N | | Mean | | SD | | SE | | Coefficient of variation | | Lower | | Upper | |
| Cortisol3.Base |  | 7 |  | 2.371 |  | 2.424 |  | 0.916 |  | 1.022 |  | 0.130 |  | 4.613 |  |
| Cortisol3.3MO |  | 7 |  | 3.071 |  | 2.267 |  | 0.857 |  | 0.738 |  | 0.975 |  | 5.168 |  |
|  | | | | | | | | | | | | | | | |

### Sample 4

| *Bayesian Paired Samples T-Test* | | | | | | | | | |
| --- | --- | --- | --- | --- | --- | --- | --- | --- | --- |
| Measure 1 | |  | | Measure 2 | | BF₋₀ | | error % | |
| Cortisol4.Base |  | - |  | Cortisol4.3MO |  | 1.909 |  | ~ 6.446×10^-6^ |  |
|  | | | | | | | | | |
| *Note.*  For all tests, the alternative hypothesis specifies that Measure 1 is less than Measure 2. For example, Cortisol1.Base is less than Cortisol1.3MO. | | | | | | | | | |

**Cortisol4.Base - Cortisol4.3MO**

**Prior and Posterior**


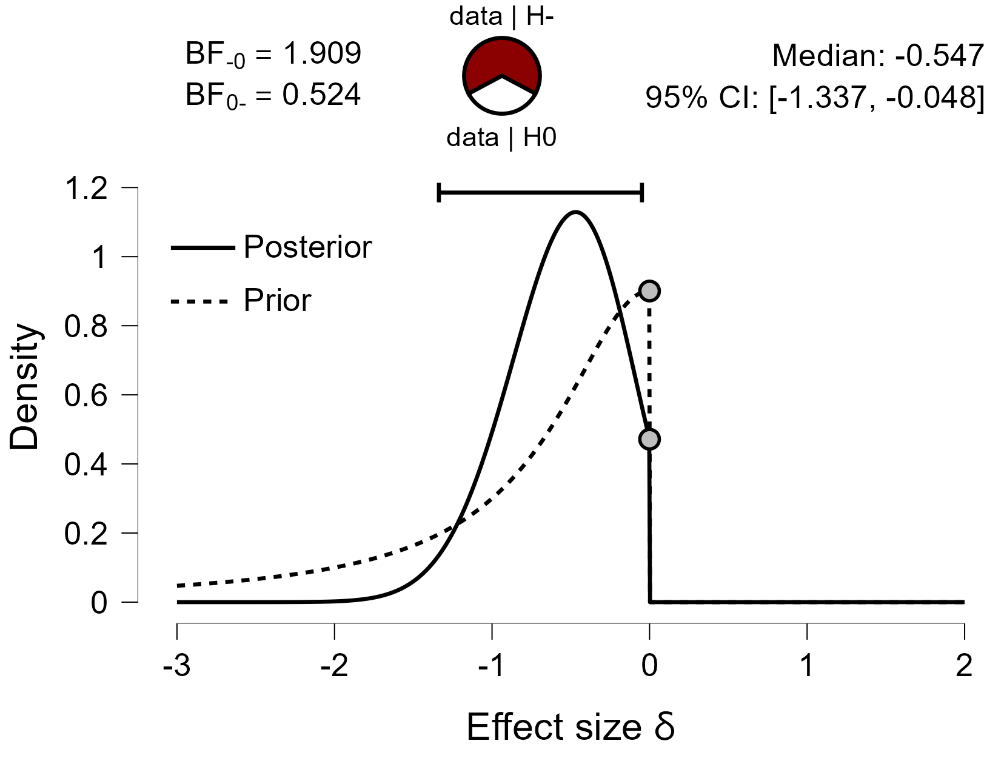


**Bayes Factor Robustness Check**


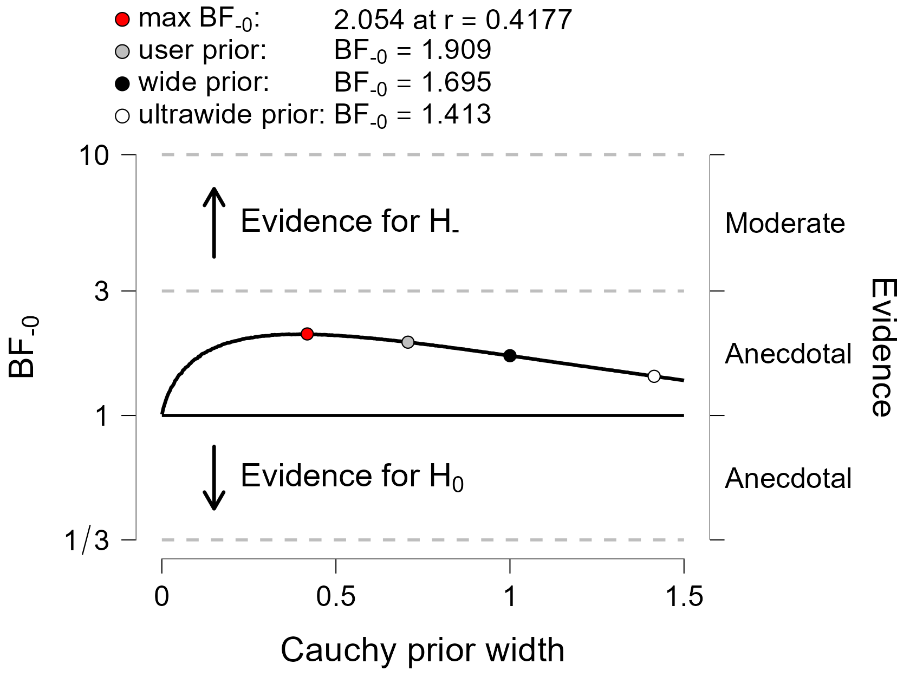


| *Descriptives* | | | | | | | | | | | | | | | |
| --- | --- | --- | --- | --- | --- | --- | --- | --- | --- | --- | --- | --- | --- | --- | --- |
|  | | | | | | | | | | | | 95% Credible Interval | | | |
|  | | N | | Mean | | SD | | SE | | Coefficient of variation | | Lower | | Upper | |
| Cortisol4.Base |  | 7 |  | 0.429 |  | 0.240 |  | 0.091 |  | 0.559 |  | 0.207 |  | 0.650 |  |
| Cortisol4.3MO |  | 7 |  | 0.893 |  | 0.834 |  | 0.315 |  | 0.935 |  | 0.121 |  | 1.665 |  |
|  | | | | | | | | | | | | | | | |

### Total cortisol mean

| *Bayesian Paired Samples T-Test* | | | | | | | | | |
| --- | --- | --- | --- | --- | --- | --- | --- | --- | --- |
| Measure 1 | |  | | Measure 2 | | BF₊₀ | | error % | |
| MeanCortAllDay.Base |  | - |  | MeanCortAllDay.3MO |  | 0.170 |  | ~ 9.394×10^-5^ |  |
|  | | | | | | | | | |
| *Note.*  For all tests, the alternative hypothesis specifies that Measure 1 is greater than Measure 2. For example, MeanCortAllDay.Base is greater than MeanCortAllDay.3MO. | | | | | | | | | |

**MeanCortAllDay.Base - MeanCortAllDay.3MO**

**Prior and Posterior**


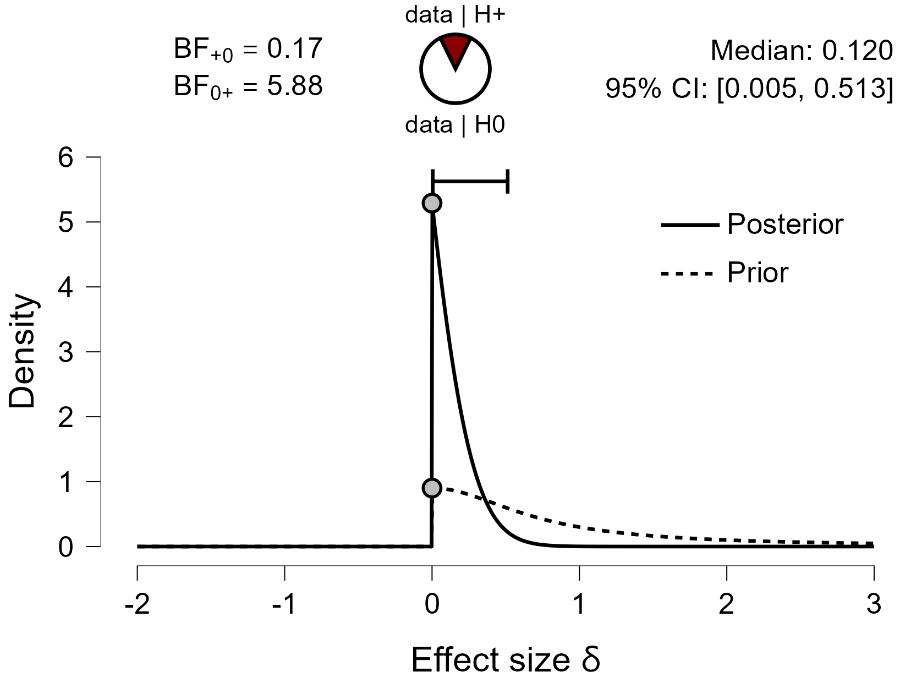


**Bayes Factor Robustness Check**


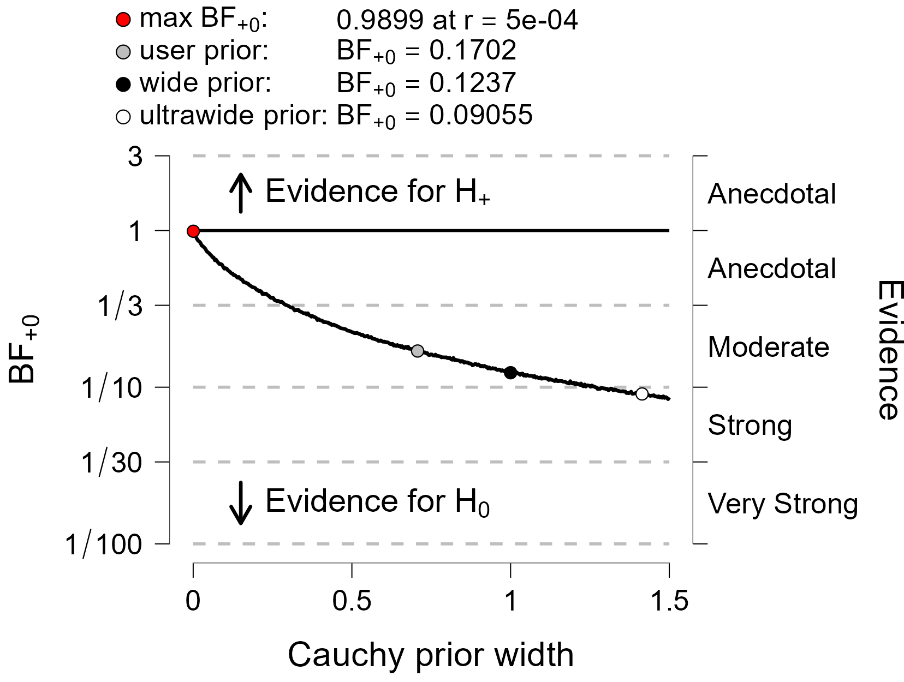


| *Descriptives* | | | | | | | | | | | | | | | |
| --- | --- | --- | --- | --- | --- | --- | --- | --- | --- | --- | --- | --- | --- | --- | --- |
|  | | | | | | | | | | | | 95% Credible Interval | | | |
|  | | N | | Mean | | SD | | SE | | Coefficient of variation | | Lower | | Upper | |
| MeanCortAllDay.Base |  | 7 |  | 2.721 |  | 0.901 |  | 0.341 |  | 0.331 |  | 1.888 |  | 3.555 |  |
| MeanCortAllDay.3MO |  | 7 |  | 3.841 |  | 1.793 |  | 0.678 |  | 0.467 |  | 2.182 |  | 5.500 |  |
|  | | | | | | | | | | | | | | | |

### AUCg

| *Bayesian Paired Samples T-Test* | | | | | | | | | |
| --- | --- | --- | --- | --- | --- | --- | --- | --- | --- |
| Measure 1 | |  | | Measure 2 | | BF₊₀ | | error % | |
| AUCg.Base |  | - |  | AUCg.3MO |  | 0.167 |  | ~ 7.740×10^-5^ |  |
|  | | | | | | | | | |
| *Note.*  For all tests, the alternative hypothesis specifies that Measure 1 is greater than Measure 2. For example, MeanCortAllDay.Base is greater than MeanCortAllDay.3MO. | | | | | | | | | |

**AUCg.Base - AUCg.3MO**

**Prior and Posterior**


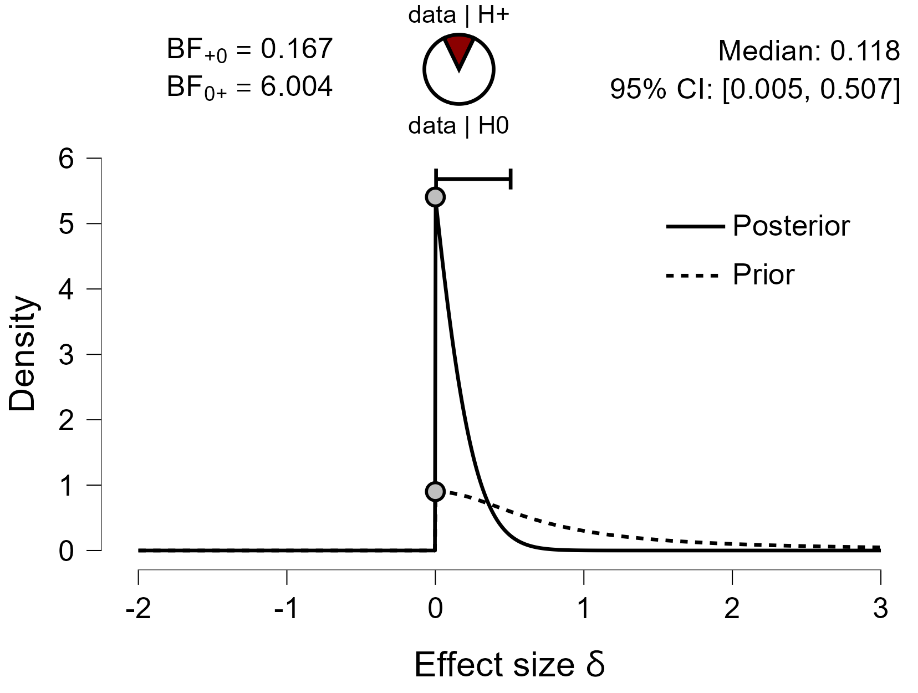


**Bayes Factor Robustness Check**


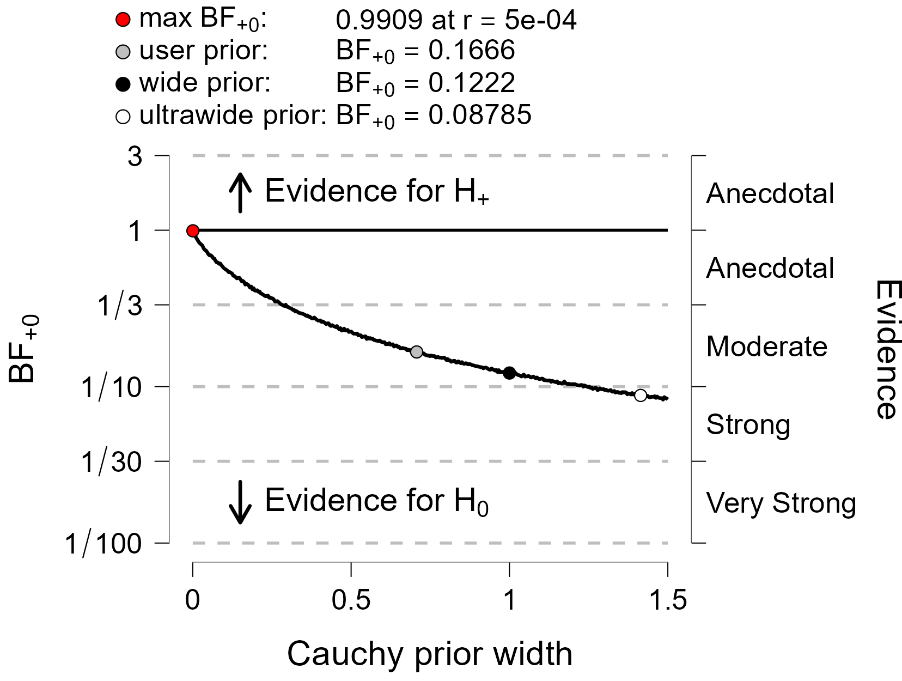


| *Descriptives* | | | | | | | | | | | | | | | |
| --- | --- | --- | --- | --- | --- | --- | --- | --- | --- | --- | --- | --- | --- | --- | --- |
|  | | | | | | | | | | | | 95% Credible Interval | | | |
|  | | N | | Mean | | SD | | SE | | Coefficient of variation | | Lower | | Upper | |
| AUCg.Base |  | 7 |  | 36.760 |  | 14.315 |  | 5.411 |  | 0.389 |  | 23.521 |  | 50.000 |  |
| AUCg.3MO |  | 7 |  | 50.628 |  | 23.271 |  | 8.796 |  | 0.460 |  | 29.106 |  | 72.150 |  |
|  | | | | | | | | | | | | | | | |

### T4 to T1 slope

| *Bayesian Paired Samples T-Test* | | | | | | | | | |
| --- | --- | --- | --- | --- | --- | --- | --- | --- | --- |
| Measure 1 | |  | | Measure 2 | | BF₊₀ | | error % | |
| CortT4toT1.Base |  | - |  | CortT4toT1.3MO |  | 0.963 |  | ~ 0.019 |  |
|  | | | | | | | | | |
| *Note.*  For all tests, the alternative hypothesis specifies that CortT4toT1.Base is greater than CortT4toT1.3MO. | | | | | | | | | |

**CortT4toT1.Base - CortT4toT1.3MO**

**Prior and Posterior**


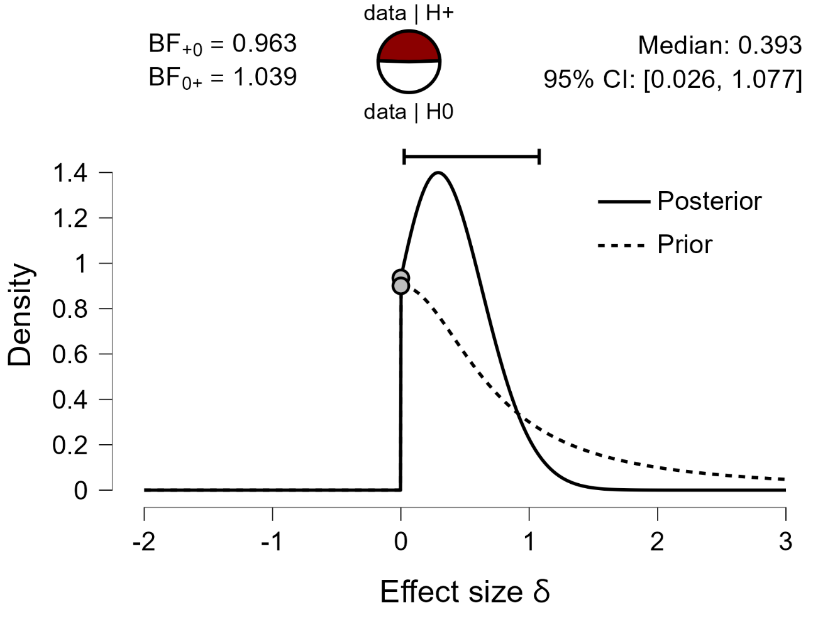


**Bayes Factor Robustness Check**


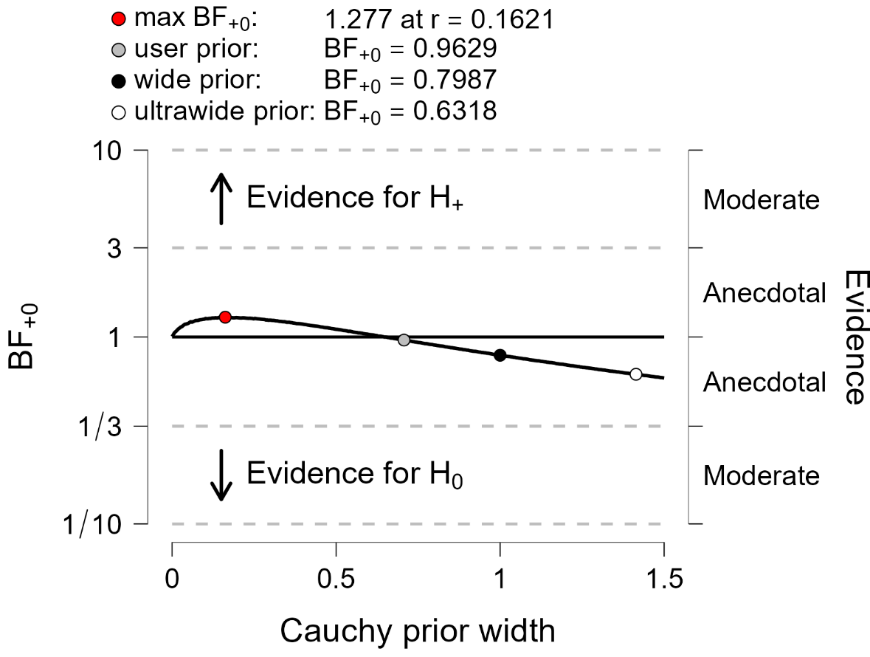


| *Descriptives* | | | | | | | | | | | | | | | |
| --- | --- | --- | --- | --- | --- | --- | --- | --- | --- | --- | --- | --- | --- | --- | --- |
|  | | | | | | | | | | | | 95% Credible Interval | | | |
|  | | N | | Mean | | SD | | SE | | Coefficient of variation | | Lower | | Upper | |
| CortT4toT1.Base |  | 7 |  | -5.914 |  | 3.208 |  | 1.213 |  | -0.542 |  | -8.881 |  | -2.947 |  |
| CortT4toT1.3MO |  | 7 |  | -8.036 |  | 4.840 |  | 1.829 |  | -0.602 |  | -12.512 |  | -3.560 |  |
|  | | | | | | | | | | | | | | | |
